# Disrupted glycosylphosphatidylinositol anchoring induces ER stress and restricts enterovirus infection

**DOI:** 10.1371/journal.ppat.1013685

**Published:** 2025-11-18

**Authors:** Shangrui Guo, Xinyu Li, Meng Xun, Yingli He, Andrew W. Tai, Hongliang Wang

**Affiliations:** 1 Key Laboratory of Tropical Translational Medicine of Ministry of Education & NHC Key Laboratory of Tropical Disease Control, Hainan Academy of Medical Sciences, Hainan Medical University, Haikou, China; 2 Department of Pathogen Biology and Immunology, Xi’an Jiaotong University Health Science Center, Xi’an, China; 3 Department of Infectious Diseases, The First Affiliated Hospital of Xi’an Jiaotong University, Xi’an, China; 4 Department of Internal Medicine & Department of Microbiology and Immunology, University of Michigan, Ann Arbor, Michigan, United States of America; 5 VA Ann Arbor Healthcare System, Ann Arbor, Michigan, United States of America; Universite de Reims Champagne-Ardenne UFR de Medecine, FRANCE

## Abstract

Many positive-sense RNA viruses, including viruses from the *Picornaviridae*, *Coronaviridae* and *Flaviviridae* family, exploit endoplasmic reticulum (ER)-derived membrane structures as sites of genome replication. Here we use a pooled CRISPR genetic screening strategy to identify glycosylphosphatidylinositol (GPI) anchor biosynthesis and transfer genes as host factors for echovirus 7 infection. In addition to supporting the biogenesis of CD55, which is a GPI anchor protein and an entry factor for some echoviruses, the GPI anchor synthesis machinery also supports several other enterovirus infections by enhancing viral replication and replication organelle biogenesis. Disruption of GPI anchor transfer machinery compromises ER integrity and causes ER stress. Consistent with these findings, ER-resident sensor, inositol-requiring protein 1α (IRE1α) is activated and regulated IRE1-dependent decay of mRNA (RIDD) is detected to reduce ER stress. Interestingly, enterovirus viral RNA, but not Hepatitis C Virus RNA, is degraded during this process due to specific sequences in the Untranslated Region (UTR). This study revealed novel links between GPI anchoring, ER stress and enterovirus infection, and illuminates new host targets for antiviral therapy.

## Author summary

Viruses are experts at hijacking cellular machinery to replicate. Many positive-sense RNA viruses, such as enteroviruses, remodel host membranes to establish replication organelles (ROs). We here discovered that a group of genes responsible for a cellular process called GPI-anchoring as critical host factors for echovirus 7 (Echo7) infection. While this pathway supports the expression of the GPI-anchored attachment factor CD55, we found it plays a more fundamental, CD55-independent role in enteroviral protein translation and the biogenesis of ROs. Mechanistically, we demonstrate that disruption of GPI anchor transfer, but not its early-stage synthesis, compromises endoplasmic reticulum (ER) integrity, inducing ER stress. This triggers the unfolded protein response (UPR), activating the IRE1α sensor and its downstream Regulated IRE1-Dependent Decay (RIDD) pathway. Intriguingly, this defense also specifically targets enteroviruses due to specific sequences in the viral untranslated regions. Our work reveals a novel link between a fundamental cellular process and the cell’s ability to fight off specific viral infections, opening new avenues for developing broad-spectrum antiviral drugs.

## Introduction

Enteroviruses of the family *Picornaviridae* include many clinically important pathogens, such as poliovirus, coxsackieviruses, echoviruses, and numbered enteroviruses including enterovirus-A71 (EV71) and enterovirus-D68 (EV68) [1]. Non-polio enterovirus infection can be associated with significant complications, including hand-foot-and-mouth disease, respiratory problems, and neurologic complications such as aseptic meningitis, encephalitis and acute flaccid paralysis [1].

Enteroviruses are non-enveloped viruses with a single-stranded, positive sense RNA genome of ~7400 nucleotides in length. The genome contains one major open reading frame (mORF) flanked by a 5’ untranslated region (UTR) and a 3’UTR. Recent studies also revealed an upstream ORF (uORF), overlapping partially with the 5’UTR, which encodes a protein to promote viral infection [2,3]. The mORF encodes a polyprotein that contains four structural proteins (VP1–4) in the P1 region and seven nonstructural proteins (2A-2C, 3A-3D) in the P2 and P3 regions [4]. Although different enteroviruses exploit different cell surface proteins as entry receptors, they share a very similar life cycle after the viral genome has been released into the cytoplasm. Once the polyprotein has been synthesized and processed to mature proteins, the nonstructural proteins (NS) rearrange intracellular membranes as sites of replication termed replication organelles (ROs) [1,5]. Electron tomography studies with poliovirus and coxsackievirus have revealed that the structures of the ROs are dynamic, with single-membrane tubules predominant at early stages of infection, followed by double membrane vesicle (DMV) and multiple-membrane structures [6,7]. These membrane structures are thought to be derived from endoplasmic reticulum (ER) or Golgi complex [5,8–10].

A large number of viruses have been shown to co-opt and remodel ER membranes to establish their replication sites. As ER is a critical organelle involved in a variety of cell metabolic and signaling functions, including lipid and glucose metabolism and calcium homeostasis [11], viral infection often disturbs ER homeostasis and causes ER stress, including accumulation of unfolded or misfolded protein, aberrant calcium regulation, and perturbation of redox status [12,13]. To cope with this, cells will activate the unfolded protein response (UPR) to reduce ER stress and restore homeostasis. The UPR consists of three signaling pathways in mammals that are mediated by three ER transmembrane proteins: inositol-requiring enzyme 1α (IRE1α), PKR-like ER kinase (PERK), and Activating Transcription Factor 6 (ATF6), the activation of which will result in reduced protein synthesis, enhanced degradation of misfolded proteins and transcriptional regulation of specific stress target genes [13,14].

While most misfolded proteins are eliminated by proteasomes in the cytosol through ER-associated degradation (ERAD), some misfolded glycosylphosphatidylinositol-anchored proteins (GPI-APs) are degraded in lysosomes [15]. GPI-APs are membrane proteins that are anchored to the outer leaflet of plasma membrane of eukaryotic cells by their GPI moiety [16,17]. The GPI anchor is assembled in the ER by a series of enzymatic reactions and covalently attached to the carboxyl terminus of proteins [17]. Human cells express more than 150 GPI-APs that function as receptors, adhesion molecules, enzymes, and transporters [16,17]. Of note, CD55 is a GPI-AP that has also been identified as a receptor for several enteroviruses, including echovirus 7, 13, 21 and several coxsackieviruses [18–20].

Here we demonstrate that knockout of GPI anchor transfer genes significantly inhibits infection of several enteroviruses, including coxsackievirus B5 (CVB5), echovirus 7 (Echo7) and EV71. However, in addition to supporting CD55 expression, which is required for Echo7 entry, these genes were also found to be required for enterovirus viral RNA (vRNA) translation and RO biogenesis. GPI anchor transfer gene depletion resulted in ER morphologic alterations. We also identified activation of the Regulated IRE1-Dependent Decay (RIDD) pathway, which led to reduced enterovirus vRNA stability. Interesting, while enterovirus vRNA is sensitive to RIDD, hepatitis C Virus (HCV) is not, and the UTR regions of enterovirus were found to be the reason of their susceptibility to GPI anchoring disruption.

## Results

### GPI anchor synthesis machinery is essential for enterovirus infection

**Fig 1:**
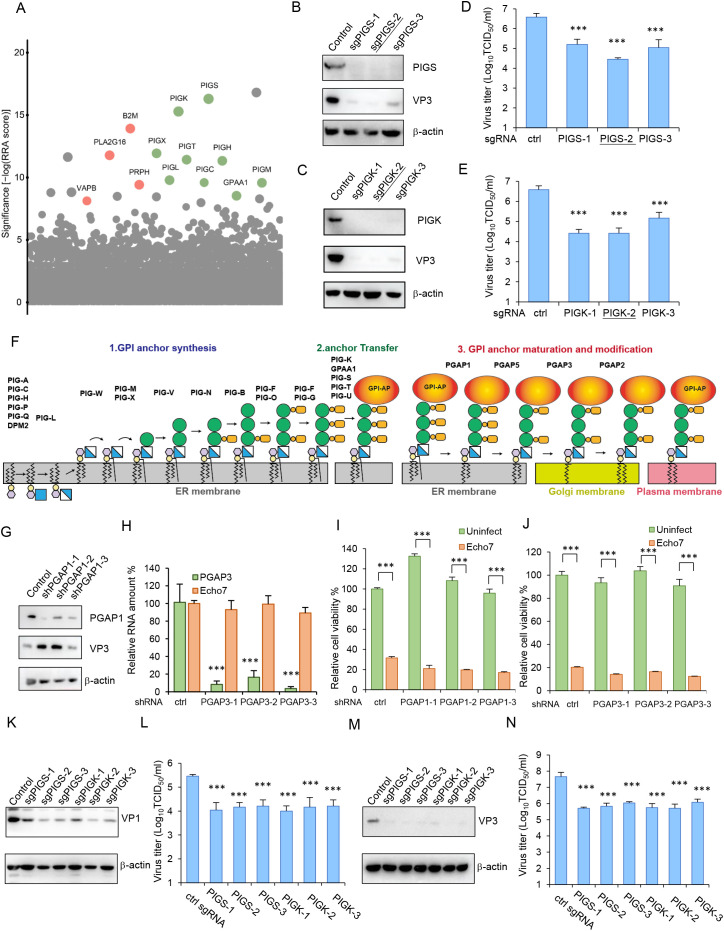
GPI-related genes are required for enterovirus infection. (A) CRISPR genetic screening for Echo7 host factors in RD cells. Each dot represents a specific gene and size corresponds to the number of sgRNAs that were enriched for each gene. Red color dots represent known enterovirus host factors. Green color dots represent GPI-related genes. (B-E) Control, PIGS-KO (B, D), or PIGK-KO (C, E) cells were infected with Echo7 virus at MOI = 1 and VP3 expression (B, C) or virus titer (D, E) was determined at 20 h.p.i. The underlined sgRNA sequences were selected for downstream knockout experiments. ***, p < 0.001, compared to control cells. (F) Diagram of GPI anchor synthesis, transfer and maturation. Enzymes catalyzing each step were listed above. (G-J) Control, PGAP1-KD (G, I), or PGAP3-KD (H, J) cells were infected with Echo7 virus at MOI = 1 and viral infection (G, H) or cell viability (I, J) was determined at 20 h.p.i. ***, p < 0.001, compared to control cells (H) or uninfected cells (I, J). (K-N) Control, PIGS-KO, or PIGK-KO cells were infected with EV71 (K, L) or CVB5 virus (M, N) at MOI = 1 and vial protein expression (K, M) or virus titer (L, N) was determined at 20 h.p.i. ***, p < 0.001, compared to control cells.

To identify host genes that are essential for enterovirus infection, we performed a whole-genome CRISPR/Cas9 screen with Echo7 infection (S1A Fig). Among the top-ranked hits were multiple molecules involved in GPI anchor synthesis, as well as the components of the transamidase enzyme complex (PIGK, PIGS, GPAA1, PIGT, PIGU) required for the transfer of GPI to the C-terminus of proteins (Fig 1A). To validate the hits from the screen, we first generated PIGS- or PIGK knockout (KO) cells through stable transduction of RD cells with lentiviral vectors encoding the sgRNA and *S. pyogenes* Cas9. The knockouts were confirmed by immunoblotting (Fig 1B-C). The cells were then infected with Echo7, and viral infection was then assessed. Significant decreases in viral titers (Fig 1D-E), viral VP3 expression (Fig 1B-C) and vRNA (S1B Fig) were observed compared to control cells. In consonance with this, while Echo7 caused marked cytopathic effect (CPE) in control cells, PIGS- or PIGK-KO cells were resistant to Echo7-induced cell death as measured by cell viability (S1C Fig). To confirm the specificity of the knockout cell lines, rescue of PIGS or PIGK expression in KO cells by exogenous lentiviral transduction of sgRNA-resistant PIGS or PIGK also restored Echo7 infection (S1D Fig). We also found that PIGT-, PIGH- or GPAA1-KO cells did not support Echo7 infection (S1E-G Fig). These data validate the results from the screen and show that multiple PIG-related genes are required for efficient Echo7 propagation.

The synthesis of GPI-APs can be divided into three steps: biosynthesis of GPI anchor, attachment of GPI anchor to proteins and GPI anchor maturation (Fig 1F). The first and second steps occur in the endoplasmic reticulum (ER), while the third occurs in ER and Golgi complex. We found that while most of the genes involved in GPI anchor synthesis and transfer were enriched in the screening, the post-GPI-attachment to proteins (PGAP) genes were not (S1 Table). Indeed, knockdown of PGAP1 or PGAP3 with shRNAs could not inhibit Echo7 infection (Fig 1G-H). Neither of them could rescue Echo7-induced cell death (Fig 1I-J), suggesting these genes are not strictly required for Echo7 infection. Taken together, the above results demonstrated that GPI anchor synthesis or transfer genes are essential host factors for Echo7 infection.

We then tested whether GPI anchor transfer machinery is also required for the infection of other enteroviruses. Control or PIGS- or PIGK-KO cells were infected with EV71 or CVB5, both of which are in the *Picornaviridae* family. Similar to Echo7, infection of EV71 or CVB5 was significantly inhibited in PIGS- or PIGK-KO cells (Figs 1K-N and S1H-I). The cell viability of PIGS- or PIGK-KO cells was significantly higher than that of control cells when they were infected with EV71 or CVB5 (S1J-K Fig). These results indicated that dependency on GPI anchor transfer machinery is shared by multiple enteroviruses.

### PIGS or PIGK supports Echo7 infection via CD55-independent mechanisms

**Fig 2:**
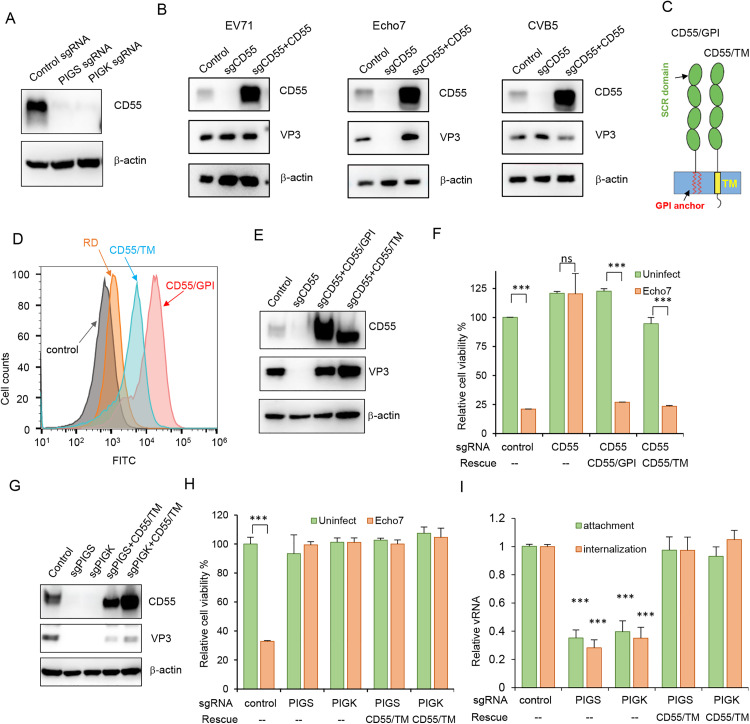
PIGS or PIGK supports Echo7 infection not just through CD55. (A) Control, PIGS- or PIGK-KO cells were immunoblotted with anti-CD55 antibody. (B) Control, CD55-KO or CD55-KO cells supplemented with sgRNA-resistant CD55 were infected with EV71, Echo7, or CVB5 and immunoblotted with indicated antibodies. (C) Diagram of GPI anchored CD55 (CD55/GPI) or chimeric CD55 with transmembrane domain (CD55/TM). (D) Surface expression of CD55 from control (orange line) or CD55-KO cells supplemented with CD55/GPI (red line) or CD55/TM (cyan line) was determined with FACS. Cells stained with isotype control antibody were used as control (grey line). (E-F) Control, CD55-KO or CD55-KO cells supplemented with CD55/GPI or CD55/TM were infected with Echo7 for 20 hrs, and CD55 and VP3 expression (E), or cell viability were determined (F). ***, p < 0.001, compared to uninfected cells. (G-H) PIGS-KO, PIGK-KO cells, or CD55/TM supplemented cells were infected with Echo7, CD55 and VP3 expression (G) or cell viability (H) was determined at 20 h.p.i. ***, p < 0.001, compared to uninfected cells. (I) Cells as described in (G) were tested for virus attachment and internalization. viral RNA was quantified by q-RT-PCR and normalized to control cells. ***, p < 0.001, compared to values in control group.

CD55 is a viral entry receptor for most Echoviruses and some serotypes of coxsackievirus B viruses (for example, CVB3). CD55 is a classic GPI-AP, the production of which depends on the GPI anchor synthesis machinery. We have shown that PIG genes are required for efficient Echo7 infection; the simplest explanation is that PIG genes support Echo7 infection through CD55 synthesis. Indeed, CD55 expression was significantly impaired in PIGS- or PIGK- KO cells (Fig 2A). However, we hypothesized that PIG genes support enterovirus infection not solely through CD55 production, because EV71 and CVB5, both of which could infect CD55-deficient cells (Figs 2B and S2A), also require PIG genes for efficient infection.

To confirm that PIG genes support Echo7 infection not solely through CD55 production, we need to evaluate the function of PIG genes without affecting functional CD55 expression. To this purpose, we designed a chimeric CD55 molecule that bears the extracellular short consensus repeats (SCR) domain and serine/threonine-rich region of CD55 linked to the CD46 transmembrane domain (CD55/TM, Fig 2C). The CD55/TM chimera is thus independent of GPI anchorage for membrane association. We then rescued CD55 expression with either sgRNA-resistant CD55 (CD55/GPI) or this chimeric CD55/TM in CD55-KO cells and flow cytometry confirmed that both molecules were expressed on the cell surface (Fig 2D). To test their function as Echo7 receptor, we infected CD55-KO cells or CD55-rescued cells with Echo7 and found that CD55-KO cells are resistant to Echo7 infection, while CD55 KO cells complemented with either CD55/GPI or CD55/TM rescued Echo7 infection (Fig 2E and 2F), demonstrating that CD55/TM, like wild type CD55, can function as an Echo7 entry receptor. In addition, we exploited the fact that GPI-APs, but not transmembrane proteins, can be cleaved by phosphatidylinositol-specific phospholipase C (PLC). We treated control, CD55 KO, or CD55 complemented cells with PLC and then tested their susceptibility to Echo7 infection. PLC treatment could ablate Echo7 infection of control cells, while CD55 KO cells treated with PLC were similarly resistant to infection as non-treated cells. CD55/GPI supplemented cells treated with PLC were resistant to Echo7 infection, consistent with GPI anchor cleavage by PLC. In contrast, CD55/TM supplemented cells still could support Echo7 infection regardless of PLC treatment (S2B Fig). These results indicate that CD55/TM can function as an Echo7 receptor independent of GPI anchorage.

We next employed this CD55/TM molecule to evaluate the role of PIGS or PIGK during Echo7 infection. PIGS- or PIGK-KO cells supplemented with CD55/TM were restored for CD55 expression, however, these cells still showed impaired infection (Fig 2G) and resistance to Echo7-induced cell death (Fig 2H). Quantification of virus attachment or internalization showed that Echo7 entered these cells as efficiently as control cells (Fig 2I). Taken together, these results showed that the GPI anchor transfer genes are necessary for efficient Echo7 infection through mechanisms that are distinct from CD55 expression and viral entry.

### PIGS or PIGK is required for optimal enterovirus protein translation

**Fig 3:**
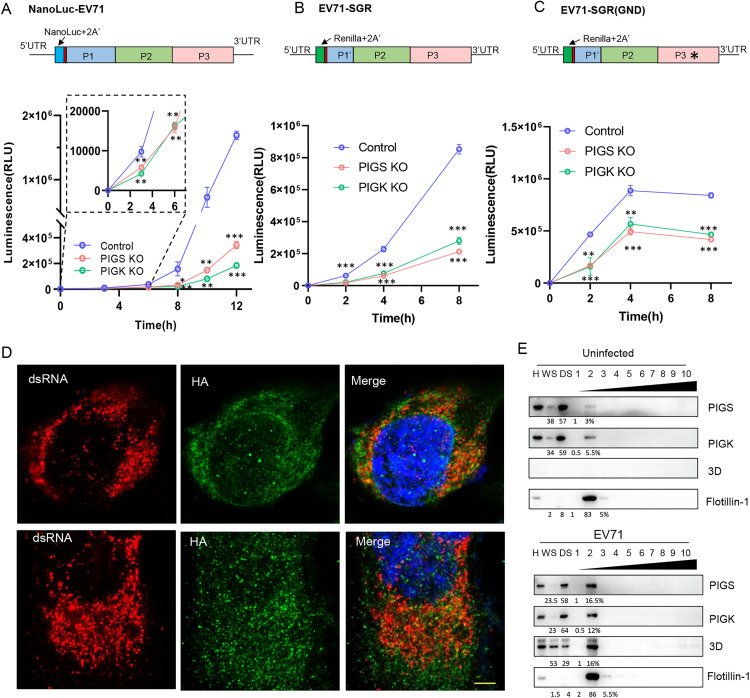
PIGS or PIGK is required for enterovirus translation. (A) Upper panel, diagram of full-length EV71 with NanoLuc reporter. Lower panel, control, PIGS-KO or PIGK-KO cells infected with NanoLuc-EV71 were monitored for luciferase activity at indicated time points. Inset, enlargement of the first 6 hrs. (B-C) Upper panels: Diagrams of EV71-SGR or EV71-SGR (GND). Lower panels: control, PIGS-KO or PIGK-KO cells were transfected with EV71-SGR (B) or EV71-SGR (GND) (C) and luciferase was monitored. (D) RD cells stably expressing PIGS-HA (upper panels) or PIGK-HA (lower panels) infected with EV71 (MOI = 1) for 8 hrs before they were immunostained with anti-HA (Green) and dsRNA (Red). Nuclei were counterstained with DAPI. Bar, 10μm. (E) Uninfected (upper) or EV71-infected (lower) RD cell homogenates (H) were centrifuged to prepare a “water soluble” supernatant (WS) or “detergent soluble” (DS) fraction. Detergent resistant membranes were then fractionated on a density gradient. Fractions (numbered in order from light to heavy) were analyzed by immunoblotting for the indicated proteins. The numbers below represent the protein mass distribution across the fractions, expressed as a percentage of the total input protein.

We next sought to find out at which specific stages GPI anchor transfer genes affect enterovirus infection. Because of the possible confounding effects of CD55 on Echo7 entry, we next used EV71, which is CD55-independent, for further characterizations. EV71 was engineered to include a NanoLuc reporter gene (Fig 3A, upper panel), which could be monitored to reflect the infection of the virus. Compared to control cells, when PIGS- or PIGK-KO cells were infected with reporter virus, these cells showed significantly and markedly reduced luciferase activity (Fig 3A, lower panels). Of note, the reduction can be detected as early as 3 h.p.i, suggesting PIG genes function at early stage of infection. As EV71 does not require CD55 for viral entry and is resistant to PLC treatment (S3A Fig), we speculated that PIGS or PIGK might affect enterovirus polyprotein translation or viral replication.

To further characterize the role of PIGS or PIGK during enterovirus infection, we next employed EV71 subgenomic replicon systems (SGRs) (Fig 3B). Due to the lack of viral structural genes, SGRs are useful tools to study vRNA translation and genome replication independently of cell entry and assembly/secretion. When PIGS- or PIGK-KO cells were transfected with EV71 SGR RNA, significantly lower luciferase was detected at all time points tested (Fig 3B). Luciferase activity was decreased as early as 2 hrs post-transfection, consistent with inhibition of viral protein translation. To confirm this, we then transfected the cells with EV71 SGRs that bear a GND point mutation at the RNA-dependent RNA polymerase catalytic site so that these SGRs could not replicate in cells (Fig 3C). We found that PIGS- or PIGK-KO cells showed lower luciferase activity even with GND SGRs, suggesting viral translation was compromised in these cells (Fig 3C). Similarly, when cells were transfected with CVB5 or Echo 7 SGR (GND), luciferase activities were also impaired in PIGS- or PIGK-KO cells (S3B-C Fig). All three viral SGRs showed lower activity, suggesting PIGS and PIGK are required for efficient viral protein translation of different enteroviruses. In contrast, when these cells were transfected with a plasmid encoding luciferase, comparable luciferase activity was detected among these cells (S3D Fig). To better study the mechanisms, we next mainly utilized EV71 for further studies.

Immunostaining of PIGS or PIGK together with dsRNA as a marker of viral replication organelles revealed partial colocalization in EV71-infected cells (Fig 3D), suggesting these molecules are in close proximity to viral replication organelles. Enterovirus ROs are enriched in cholesterol and therefore they are insoluble in cold detergent solutions [21–23]. The detergent-resistant membrane (DRM) fraction of EV71 infected cells were isolated and subjected to gradient centrifugation. Fig 3E showed that part of viral NS protein 3D co-fractioned with PIGS, PIGK together with the DRM marker flotillin-1. Collectively, these results suggest that PIGS and PIGK are required for optimal enterovirus protein translation. The underlying mechanism for this requirement will be investigated in the following sections.

### PIGS or PIGK is required for enterovirus replication organelle formation

**Fig 4:**
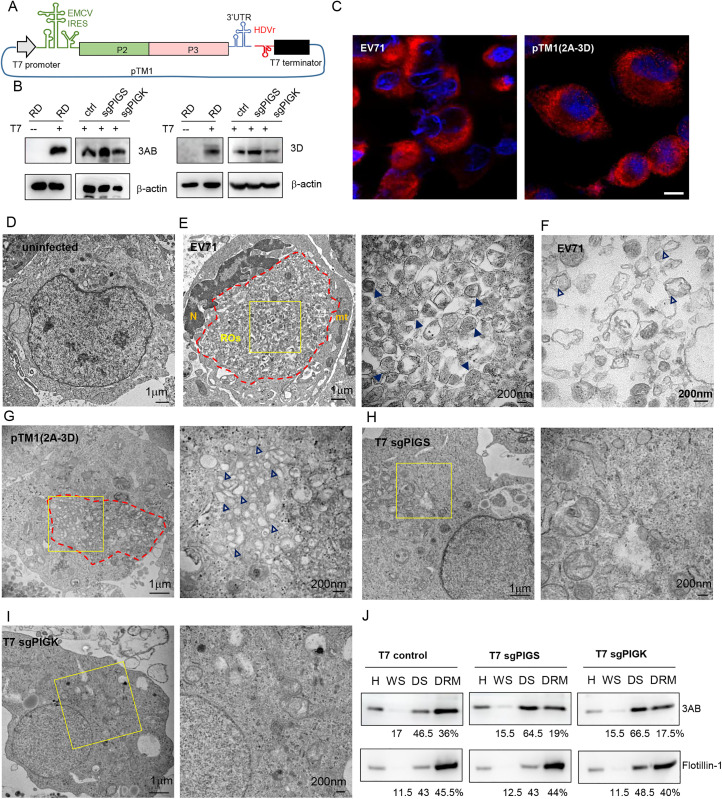
PIGS or PIGK is required for enterovirus RO biogenesis. (A) Diagram of pTM1 (2A-3D). The P2 and P3 regions of EV71 genome together with EV71 3’UTR were put downstream of T7 promoter and EMCV IRES, followed by HDV ribozyme and T7 terminator. (B) RD cells, PIGS-KO or PIGK-KO cells stably transduced with T7 polymerase were transfected with pTM1 (2A-3D) and immunoblotted with anti-3AB or anti-3D antibody.β-actin was used as loading control. (C) RD cells infected with EV71 were immunostained with anti-3AB antibody with nuclei counterstaining (left panel). RD/T7 cells were transfected with pTM1 (2A-3D) and immunostained with anti-3AB antibody (right panel). Bar, 10μm. (D) Representative electron microscopy image of uninfected RD cell. (E) Representative electron microscopy images of EV71 induced membrane alterations. Area with extensive membrane alterations was circled with red dashed lines. Enlargement of the boxed area was shown on right. Arrowheads indicate electron-dense membrane vesicles. (F) Representative image of EV71 infected RD cells with membrane vesicles. Empty arrowheads indicate electron-translucent DMVs. (G) Representative images of RD/T7 cells transfected pTM1 (2A-3D). Red dashed lines indicate area of membrane alterations. Enlargement of the boxed area was shown on the right. Empty arrowheads indicate electron-translucent DMVs. (H-I) PIGS-KO (H) or PIGK-KO cells (I) stably expressing T7 polymerase were transfected with pTM1 (2A-3D) and electron microscopy images were shown. Enlargement of the boxed areas were shown on the right. (J) Cells described in (B) were transfected with pTM1 (2A-3D) and subjected to fractionation. 3AB or flotillin-1 proteins in each faction was determined with immunoblotting. The numbers below represent the protein mass distribution across the fractions, expressed as a percentage of the total input protein.

Enterovirus ROs are modified by viral NS from host intracellular membranes. Since both PIG genes are important for enterovirus translation, we speculate that they are also required for RO formation. Because enterovirus polyprotein translation is coupled to RNA replication, inhibition of host factors affecting viral replication will inevitably lead to decreased polyprotein translation in authentic replication systems. For this purpose, we designed a replication-independent polyprotein expression system to study enterovirus RO biogenesis. In this system, the EV71 NS coding region together with the 3’UTR region were placed downstream of the T7 promoter and an EMCV IRES followed by hepatitis D virus ribozyme sequence (pTM1 (2A-3D); Fig 4A) [24]. Viral NS immunoblotting showed that transfection of this construct into RD cells stably expressing T7 RNA polymerase (RD/T7) led to viral NS expression, while RD cells lacking T7 RNA polymerase did not generate any 3AB or 3D expression (Fig 4B). More importantly, the expression of 3AB or 3D were comparable between the control and PIGS- or PIGK-KO cells (Fig 4B), suggesting the EMCV-IRES driven viral NS expression was not sensitive to PIGS- or PIGK-KO.

RD/T7 cells transfected with pTM1 (2A-3D) displayed a reticular staining of 3AB surrounding the nucleus, extended throughout the cytoplasm (Fig 4C, right panel), which is characteristic of enterovirus ROs staining, similar to those seen in cells infected with EV71 virus (Fig 4C, left panel). We then employed this system to examine the biogenesis of ROs with transmission electron microscopy (TEM). Compared to uninfected cells (Fig 4D), EV71 infection caused dramatic membrane alterations with clusters of membrane structures occupying large area of the perinuclear cytoplasm (Fig 4E). These clusters contained packed membrane vesicles, most of which contain electron-dense interior (Fig 4E, right panel), but some of them are electron-translucent (Fig 4F), perhaps reflecting different stages of viral infection. Higher magnification images confirmed that most of them are closed single- and double-membrane vesicles (SMVs and DMVs) (Fig 4E, right panel). Similarly, dramatic membrane alterations were also observed in RD/T7 cells transfected with pTM1 (2A-3D) (Fig 4G), with packed SMVs and DMVs in the cytoplasm (Fig 4G, right panel, S4A Fig). However, most of these DMVs seemed more electron-translucent compared to EV71-induced vesicles, perhaps due to the lack of viral structural genes in the transfection system and thus no dense granules were assembled. In contrast, membrane alterations were much less conspicuous in PIGS- or PIGK-KO cells transfected with pTM1 (2A-3D) (Figs 4H-I, S4B, and S4D). Occasionally, multiple membrane vesicles were observed (S4C and S4E Fig). Quantification of these membrane vesicles showed that there were significant reductions of vesicle numbers in the KO cells (S4F Fig).

Although viral NS protein expression was comparable, the significantly reduced number of viral ROs suggests that some of the expressed NS proteins are unable to induce RO formation. In consonance with this, we found lower 3AB protein enrichment in the DRM fractions of PIGS- or PIGK-knockout cells compared to control cells (Fig 4J). Notably, the 3A protein is a membrane-targeting protein, and it has been reported to be responsible for the reorganization of membranes in the formation of ROs [10,25]. Taken together, these results suggest that these PIG genes were required for the biogenesis of ROs.

### PIGS or PIGK is essential to maintain ER integrity

**Fig 5:**
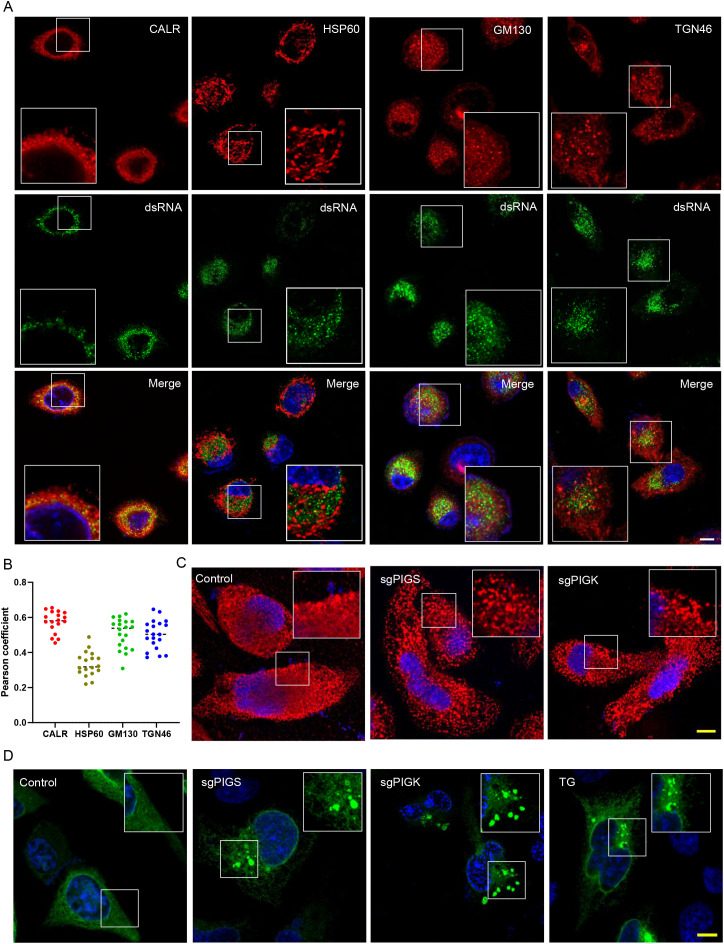
PIGS or PIGK maintains ER integrity. (A) RD cells were infected with EV71 at MOI = 1 for 6 hrs and they were immunostained with anti-CALR, GM130 or TGN46 together with anti-dsRNA. Nuclei were counterstained with DAPI. Bar, 10μm. (B) Quantification of pearson’s correlation coefficient of CALR, HSP60, GM130 or TGN46 with dsRNA. Each data point corresponds to the coefficient derived from a single image. (C) Control, PIGS- or PIGK-KO cells were immunostained with CALR with nuclei counterstaining. Bar, 10μm. (D) Control, PIGS- or PIGK-KO cells were transfected with Sec61β-GFP and nuclei were counterstained with DAPI. 0.5μM TG treatment for 6 hrs was used to induce ER whorl formation. Bar, 10μm.

ROs from several enteroviruses have been reported to originate from the ER or trans-Golgi network [8*–*10]. We next set out to see whether EV71-induced viral ROs colocalized with ER or Golgi complex. RD cells infected with EV71 were immunostained with dsRNA and ER marker calreticulin (CALR), cis-Golgi Marker GM130, trans-Golgi Marker TGN46 or mitochondria marker HSP60 (Fig 5A). At 6 h.p.i, Pearson’s coefficient showed that dsRNA had highest colocalization with CALR (r = 0.575), followed by Golgi marker GM130 and TGN46 (r = 0.508 and 0.502, respectively). dsRNA had little colocalization with HSP60 (Fig 5B, r = 0.334). These results suggest that EV71 virus ROs are associated with ER and Golgi, and are consistent with the notion that most enteroviruses ROs are derived from these organelles.

Since most of the PIG genes localized to ER, we next wanted to know whether the depletion of these genes affects the morphology of ER. For this purpose, we stained control or PIGS- or PIGK-KO cells with ER marker CALR. In control cells, CALR exhibited fine, reticular staining pattern throughout the cytoplasm (Fig 5C). In PIGS- or PIGK-KO cells, however, CALR exhibited a more clustered pattern, although it also extended throughout the cytoplasm (Fig 5C). This phenomenon was confirmed when cells were transfected with a plasmid encoding GFP-tagged Sec61β, another well-established ER marker (Fig 5D). Large Sec61β-positive structures were also observed in PIGS- or PIGK-KO cells, resembling the ER whorls observed [26] when cells were treated with thapsigargin (TG) (Fig 5D). In contrast, when these cells were stained for GM130 or TGN46, no significant changes were observed compared to control cells (S5A Fig).

Enteroviruses have been reported to utilize lipid droplets (LDs) as source of lipid metabolism to promote RO biogenesis [27] and LDs are believed to emerge from ER. We next evaluated whether lipid droplets biogenesis is compromised in PIGS- or PIGK-KO cells. Lipid droplets were stained with Bodipy 493/503 and comparable amounts of LDs were observed among different cells with or without oleic acid treatment (S5B-C Fig). Similarly, no significant difference was observed when the total cellular lipids were quantified (S5D Fig). Taken together, these results suggest that PIGS- or PIGK-KO cells have altered ER morphology and that this may then inhibit enterovirus RO biogenesis.

### Disruption of PIGS or PIGK leads to ER stress and viral RNA degradation

**Fig 6:**
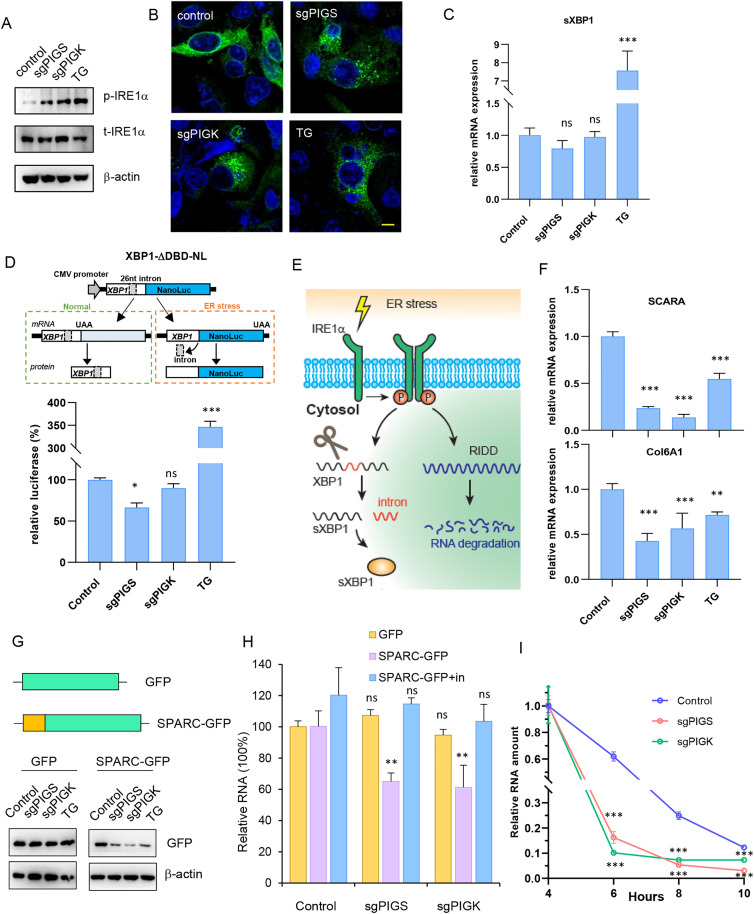
Enterovirus RNA is degraded via RIDD pathway. (A-C) Control, PIGS-, PIGK-KO cells were immunoblotted with indicated antibodies (A), transfected with IRE1-GFP and evaluated with confocal microscopy (B) or tested for spliced XBP1 expression with q-RT-PCR (C). Control cells treated with 0.15μM TG for 6 hrs were used as positive control for ER stress. ns, not significant; ***, p < 0.001, compared to control. (D) Upper panel, diagram of IRE1-splicing dependent NanoLuc expression system (XBP1-ΔDBD-NL). Lower panel, indicated cells were transfected with XBP1-ΔDBD-NL and luciferase was monitored and normalized to control cells. ns, not significant; *, p < 0.05; ***, p < 0.001, compared to control. (E) Diagram of IRE1α activation pathways. IRE1α-mediated XBP1 alternative splicing and RIDD pathways were shown. (F) SCRAR, Col6A1 expression in control, PIGS- or PIGK-KO cells were determined with q-RT-PCR. **, p < 0.01; ***, p < 0.001, compared to control group. (G) GFP and SPARC-GFP expression in control, PIGS- or PIGK-KO cells determined with immunoblotting. (H) GFP, or SPARC-GFP expression with or without 20μM 4μ8C treatment was determined with q-RT-PCR in control, PIGS- or PIGK-KO cells. in, 4μ8C inhibitor. ns, not significant; **, p < 0.01, compared to values in control group. (I) Control, PIGS- or PIGK-KO cells were transfected with EV71-SGR (GND) RNA and remaining vRNA were determined 4, 6, 8 or 10 hrs later. Values were normalized to 4 hr data in corresponding group. ***, p < 0.001, compared to control group.

The changes in ER morphology in PIGS- or PIGK-KO cells suggested the possibility of ER stress in these cells. The adaptive response to ER stress is the UPR, which is initiated by three ER transmembrane proteins: IRE1α, PERK, and ATF6 [13]. Upon sensing the presence of unfolded or misfolded proteins, IRE1 dimerizes and autophosphorylates to become active [28]. Consistent with this, we found IRE1α phosphorylation was upregulated in PIGS- or PIGK-KO cells, comparable to TG treatment (Fig 6A). In addition, cells transfected with a construct expressing IRE1α-GFP [29] were evaluated for IRE1α oligomerization. Discrete IRE1α foci were observed with TG treatment as well as in PIGS- or PIGK-KO cells (Fig 6B), suggesting that IRE1α oligomerization and activation is associated with PIGS or PIGK deficiency. Activated IRE1α excises a 26-nt intron from the XBP1 mRNA [13], generating sXBP1, which translocates into the nucleus and upregulates UPR target genes [28]. In PIGS- or PIGK-KO cells, however, XBP1 splicing was not upregulated as determined by q-RT-PCR (Fig 6C). Similarly, the expression of an XBP-1 splicing-dependent luciferase reporter (Fig 6D) was also not upregulated in PIGS- or PIGK-KO cells, suggesting that the pathways downstream of IRE1α activation differ in PIGS- or PIGK-KO cells compared to TG induced ER stress.

IRE1 activation also mediates the cleavage and degradation of mRNAs encoding ER-localized/secretory proteins, an activity termed Regulated IRE1-Dependent Decay (RIDD) (Fig 6E) [30,31]. Interestingly, several RIDD target genes, such as SCRAR and Col6A1 were downregulated in PIGS or PIGK knockout cells as assayed by q-RT-PCR (Fig 6F), suggesting that IRE1 activation in PIGS- or PIGK-KO cells can induce RIDD. The known substrates of RIDD are ER-localized mRNAs or mRNAs encoding secretory proteins. Consistent with this, cytoplasmically expressed GFP was not degraded with TG treatment or PIGS/PIGK KO, while GFP fused with the ER anchor of SPARC, which is a known substrate of RIDD [30], was significantly down-regulated in PIGS- or PIGK-KO cells (Fig 6G) and this downregulation was occurred at the transcription level (Fig 6H). More importantly, 4μ8C, which is an IRE1 endonuclease activity inhibitor that would not inhibit its kinase activity, rescued SPARC-GFP mRNA expression (Fig 6H), suggesting the mRNA degradation was mediated by IRE1’s endonuclease activity and that RIDD was activated in these cells.

Enteroviruses employ cellular translation machinery to produce viral proteins and viral replication occurs on ER- and Golgi-derived membrane structures. Since the RIDD pathway was activated in PIGS- or PIGK-KO cells, we speculated viral RNA, which was enriched in ER, could also be degraded to reduce ER load. We then measured viral RNA stability in these cells. To avoid any effect the cells may have on viral entry or replication, this experiment was carried out by transfecting replicase-dead EV71 viral RNA (EV71 SGR GND) into cells and the amount of viral RNA was quantified at different time points. We found that vRNA stability was significantly reduced in PIGS- or PIGK-KO cells (Fig 6I), and 4μ8C partially rescued viral infection in PIGS- or PIGK-KO cells (S6G Fig). These results suggest that viral infection declines in PIGS- or PIGK-KO cells are likely due to the activation of RIDD in these cells.

The activation of PERK and the ATF6 pathway was also investigated. Immunoblotting showed that PERK phosphorylation, eIF2α phosphorylation and ATF4 induction was minimally increased in PIGS- or PIGK-KO cells (S6A Fig). Transcriptional evaluation of CHOP and GADD34 are not up-regulated either in these cells (S6B-C Fig). Similarly, transcriptional evaluation of BIP or PID4A of the ATF6 branch was not activated (S6D-E Fig). Cells transfected with a luciferase driven by 5 × ATF6 binding sites [32] generated luciferase similar to that of control cells (S6F Fig). Taken together, these results suggested that PERK or ATF6 branches of the UPR were not activated in PIGS- or PIGK-KO cells.

### Activation of RIDD pathway requires late-stage GPI anchor precursors

**Fig 7:**
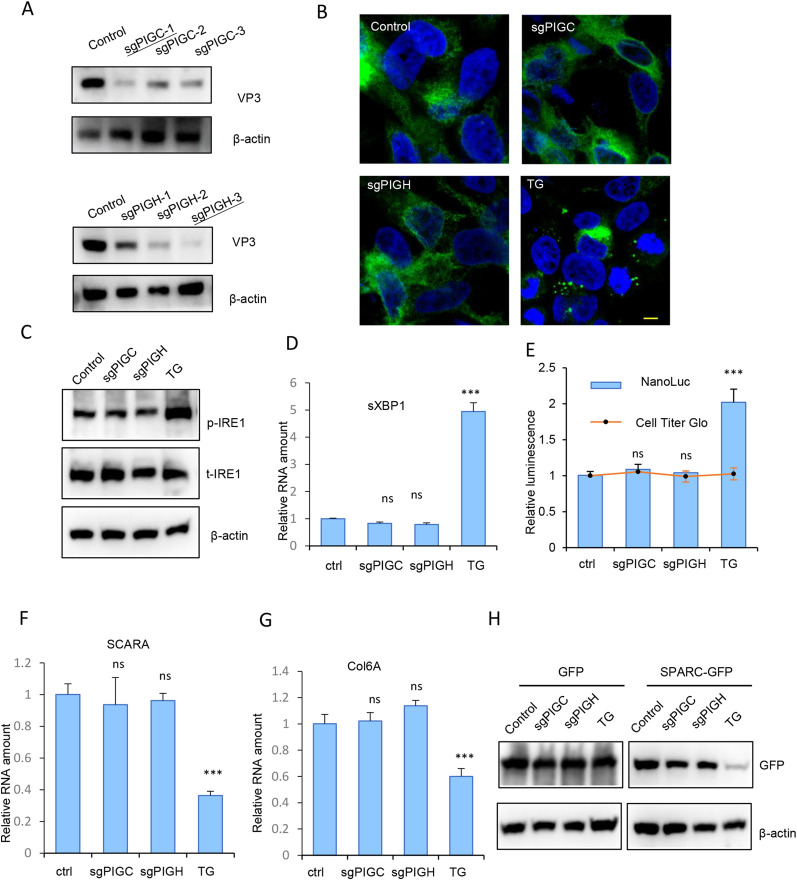
Knockout of PIGC or PIGH does not activate RIDD. (A) Control, PIGC KO (upper panels), or PIGH KO (lower panels) cells were infected with Echo7 virus at MOI = 1 for 20 hrs and viral VP3 expression was detected with immunoblotting. sgRNAs underlined were chosen for downstream experiments. (B) Control, PIGC KO, or PIGH KO cells transfected with IRE1-GFP and evaluated with confocal microscopy. TG was used as a positive control. Bar, 10μm. (C-D) Control, PIGC KO, or PIGH KO cells were immunoblotted with indicated antibodies (C) or tested for spliced XBP1 expression with q-RT-PCR (D). ns, not significant; ***, p < 0.001, compared to values in control group. (E) Indicated cells were transfected with XBP1-ΔDBD-NL and luciferase was measured. Cell viability was measured with Cell Titer Glo. ns, not significant; ***, p < 0.001, compared to values in control group. (F-G) SCARA (F) or Col6A1 (G) expression in indicated cells were determined with q-RT-PCR. ns, not significant; ***, p < 0.001, compared to values in control group. (H) Indicated cells were transfected with GFP or SPARC-GFP and the expression was determined with immunoblotting.

Both PIGS and PIGK are subunits of the GPI transamidase complex, which catalyzes the transfer of GPI-anchor to proteins bearing a carboxyl-terminal GPI attachment signal [17]. Therefore, knockout of PIGS or PIGK could lead to the accumulation of both lipid moiety (GPI anchor and precursors) and protein moiety (unprocessed, immature proteins). We next wanted to know whether the activation of RIDD pathway during PIGS- or PIGK-knockout was due to the accumulation of lipid or protein moiety. For this purpose, we chose to inhibit PIGC or PIGH expression, both of which are involved in the first step of GPI-anchor synthesis (Fig 1F) and the successful inhibition of these targets would abolish the synthesis of GPI-anchor. The successful knockout of PIGC and PIGH was confirmed by impaired Echo7 virus infection (Fig 7A) and sequencing of the targeted genomic loci (S7A Fig). However, IRE1α activation or sXBP1 upregulation was not observed in these cells (Fig 7B-E). In addition, RIDD target genes, SCARA or Col6A were not downregulated (Fig 7F-G) and SPARC-GFP expressed at levels comparable to control cells (Fig 7H), suggesting that inhibiting GPI anchor synthesis at its initial stages does not activate the RIDD pathway. Similarly, when knocking out PIGB or PIGO (S7B-C Fig), which functions at intermediate stages of the pathway, no RIDD activation was observed (S7D-J Fig). Taken together, these results suggest that activation of the RIDD pathway requires late-stage GPI anchor precursors.

### Enterovirus infection activates PERK pathway during late stage of infection

**Fig 8:**
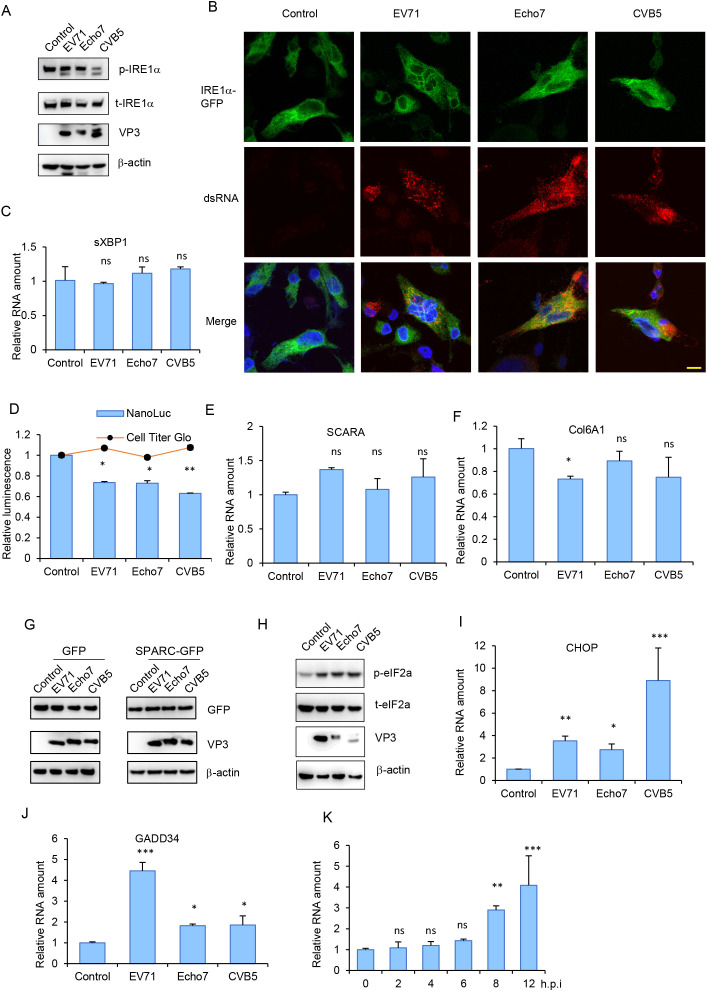
Enterovirus infection activates PERK pathway during late stage of infection. (A) RD cells infected with EV71, Echo7 or CVB5 (MOI = 1) were immunoblotted with indicated antibodies. (B) RD cells transfected with IRE1-GFP were infected with EV71, Echo7 or CVB5 (MOI = 1) and immunostained with dsRNA (red). Nuclei were counterstained with DAPI. Bar, 10μm. (C) RD cells infected with EV71, Echo7 or CVB5 were tested for spliced XBP1 expression with q-RT-PCR. ns, not significant, compared to uninfected cells. (D) RD cells transfected with XBP1-ΔDBD-NL were infected with EV71, Echo7 or CVB5 and luciferase was determined. Cell viability was measured with Cell Titer Glo. *, p < 0.05; **, p < 0.01, compared to uninfected cells. (E-F) RD cells infected with EV71, Echo7 or CVB5 were tested for SCRAR (E) or Col6A1 (F) expression with q-RT-PCR. ns, not significant; *, p < 0.05, compared to uninfected cells. (G) RD cells transfected GFP or SPARC-GFP were infected with EV71, Echo7 or CVB5 and immunoblotted with indicated antibodies. (H) RD cells infected with EV71, Echo7 or CVB5 were immunoblotted with indicated antibodies. (I-J) RD cells infected with EV71, Echo7 or CVB5 were tested for CHOP (I) or GADD34 (J) expression with q-RT-PCR. *, p < 0.05; **, p < 0.01; ***, p < 0.001, compared to uninfected cells. (K) RD cells infected with CVB5 for indicated time were tested for CHOP expression with q-RT-PCR. ns, not significant; **, p < 0.01; ***, p < 0.001, compared to values in time = 0 h.

Given that many viruses activate UPR during infection [33], we next investigated whether enteroviruses also activate UPR. By using the methods described above, we found no evidence of IRE1α activation or spliced XBP1 upregulation during EV71, Echo7 or CVB5 infection (Figs 8A-D and S8A). Furthermore, the RIDD target genes, such as, *SCARA* and *Col6A1*, as well as the transfected *SPARC-GFP* expression remained unaffected (Figs 8E-G and S8B), confirming IRE1 pathway was not activated. However, we did observe significant upregulation of eIF2α phosphorylation, indicative of PERK pathway activation, during enterovirus infection (Fig 8H). Consistent with this, downstream PERK target genes like *CHOP* and *GADD34* were also upregulated (Fig 8I-J), suggesting the activation of this pathway. Time-course analysis of CHOP expression revealed upregulation beginning at 8 or 12 h.p.i (Fig 8K), suggesting this pathway was mainly activated relatively late in the viral life cycle. The ATF6 pathway was also tested and no activation was detected (S8C-E Fig).

### Enterovirus UTR regions are sensitive to GPI anchor transfer machinery disruption

In addition to enteroviruses, many other RNA viruses replicate on ER-derived membranes, for example, flaviviruses and coronaviruses [34,35]. Hepatitis C Virus (HCV) and enteroviruses have great similarities in terms of viral replication. First, both HCV and enteroviruses replicate on ER-derived, positively curved membranes, comprising single-, double- and multiple membrane structures [34,35]. Second, ROs of these viruses all have specialized lipid composition, which are maintained by PI4 kinases and lipid transfer proteins [23,36,37].

**Fig 9:**
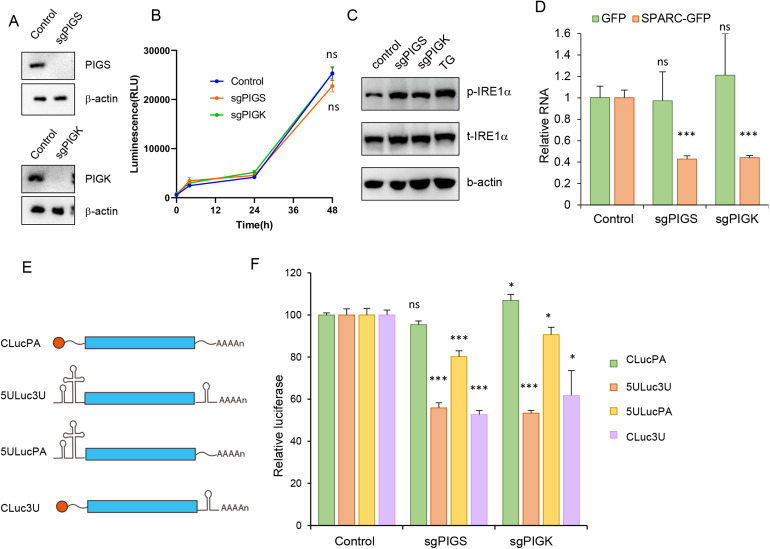
The UTRs are responsible for enterovirus sensitivity to ER stress. (A) Control, PIGS- or PIGK-KO Huh 7 cells were immunoblotted with indicated antibodies. (B) Control, PIGS- or PIGK-KO Huh 7 cells were transfected with HCV-SGR and luciferase activity was monitored at indicated time points. ns, not significant, compared to control cells. (C) Control, PIGS- or PIGK-KO Huh 7 cells were immunoblotted with IRE1α antibodies. (D) Control, PIGS- or PIGK-KO Huh 7 cells were transfected with GFP or SPARC-GFP and relative mRNA was determined with q-RT-PCR. ns, not significant; ***, p < 0.001, compared to values in control group. (E) Diagrams of luciferase expressing RNA with different combination of 5’- or 3’-UTR. (F) Expression of different luciferase RNAs in control, PIGS- or PIGK KO cells. Values were normalized to control groups. ns, not significant; *, p < 0.05; ***, p < 0.001, compared to values in control group.

We next want to test whether HCV infection was also compromised in PIGS- or PIGK-KO cells. Huh 7 cells stably transduced with PIGS- or PIGK-sgRNA lentivirus were first immunoblotted for target protein expression (Fig 9A) and then transfected with RNA encoding an HCV SGR with luciferase reporter [38]. As can be seen from Fig 9B, comparable luciferase activities were observed for control and PIGS- or PIGK-KO cells, indicating that the replication of HCV viral RNA was not affected by PIGS or PIGK KO. However, IRE1 phosphorylation and RIDD were also detected in PIGS- or PIGK-KO Huh 7 cells (Fig 9C-D), consistent with the presence of ER stress in these cells. These results suggested that although HCV also replicates on ER-derived membranes, it was not as sensitive to ER stress as enteroviruses.

While the EV71 SGR bearing luciferase was degraded in PIGS- or PIGK-KO cells, the expression of luciferase reporter gene, as well as the cytoplasmic GFP, was not inhibited in these cells (S3D and S9A-B Figs). We speculated that some cis-elements in the EV71 viral RNA was essential for its localization and recognition by the RIDD machinery for degradation. The untranslated regions (UTR) of viral genome are essential for viral replication and translation [39]. To elucidate the role of 5’UTR and 3’UTR of viral genome for vRNA expression, we designed four luciferase reporters with different UTR region (Fig 9E): ① luciferase with 5’ cap and 3’ poly A tail (CLucPA); ② luciferase with 5’UTR and 3’UTR from EV71 viral genome (5ULuc3U); ③ luciferase with EV71 5’UTR and poly A tail (5ULucPA); ④ luciferase with 5’ cap and EV71 3’UTR (CLuc3U). All constructs were *in vitro* transcribed, modified accordingly and transfected into control or PIGS- or PIGK-KO cells. As shown in Fig 9F, luciferase expression from 5ULuc3U was significantly downregulated compared to CLucPA in PIGS- or PIGK-KO cells. In addition, luciferase from CLuc3U was almost as low as 5ULuc3U, while the luciferase of 5ULucPA was also compromised. Consistent with this, when RD cells overexpressing an endoribonuclease dead mutant of IRE1 (IRE1 K907A) [40] and transfected with luciferase RNA were subjected to RNA immunoprecipitation (RIP), more 5ULuc3U RNA was precipitated compared to CLucPA (S9C-F Fig). These results suggested that the UTR regions were essential for vRNA subjection to RIDD.

## Discussion

The ER is the largest organelle in the cell and is the primary site of protein synthesis and folding, lipid synthesis and metabolism, and calcium storage [11]. Accumulation of misfolded proteins [13] as well as changes in lipid metabolism [41] will cause ER stress, which initiates UPR. Importantly, mechanisms of UPR activation caused by lipid perturbation are different from those resulted from misfolded proteins [41]. GPI-APs are specialized integral membrane proteins anchored by a GPI lipid moiety. The conserved GPI moiety is synthesized and then transferred to the C-terminus of proteins by more than 20 enzymes on the ER membrane [17]. These immature GPI-APs will undergo several remodeling steps in the ER and the Golgi apparatus before they are transported to the plasma membrane. As GPI-APs comprise both protein and lipid moieties, errors during GPI-AP synthesis might activate various responses in the cell. In line with this hypothesis, we here demonstrated that disruption of GPI anchor transfer led to morphology changes in the ER and ER stress. A similar phenomenon was also observed when Gwt1, a critical acyltransferase required for the biosynthesis of fungal GPI anchors was inhibited [42]. Furthermore, we also observed that blocking GPI anchor transfer triggered RIDD pathway activation, whereas inhibiting synthesis at the initial or intermediate stages did not. Therefore, our findings indicate that although blocking anchor transfer causes the accumulation of both protein and lipid moieties, RIDD activation is specifically triggered by the accumulating lipid component, most likely the late-stage GPI anchor precursors or the mature GPI anchor itself. Conversely, it has been reported that misfolded GPI-APs, complete with their GPI anchor attachment, will transiently access the plasma membrane before being rapidly targeted to lysosomes for degradation [15,43]. Therefore, distinct mechanisms are engaged to relieve ER stress at different steps of GPI-AP biogenesis.

Cellular UPR in mammals is regulated by three master regulators, IRE1α, PERK and ATF6, which mediate transcriptional or translational mechanisms to alleviate stress [13]. Here we found that IRE1 pathway was activated when GPI anchor transfer was abolished. However, instead of activating the canonical transcriptional factor, XBP1, GPI synthesis disruption led to activation of RIDD. RIDD has been documented to degrade a specific subset of mRNAs that encoding proteins traffic through the ER but are not directly involved in ER function [30]. Enteroviral RNA, which is likely to exhibit features of RIDD target mRNA, replicates on ER-derived membrane structures and was degraded when GPI synthesis pathway was inhibited. Like other positive-sense RNA viruses, enterovirus vRNA is used for either translation or packaging, but not both simultaneously. Consequently, vRNA degradation via RIDD inevitably impairs viral translation and subsequent replication. Furthermore, since viral nonstructural (NS) proteins drive the rearrangement of intracellular membranes to form ROs, impaired NS protein translation inhibits RO biogenesis. This explains our observation of defective vRNA translation and RO biogenesis in PIGS- and PIGK-knockout cells (Fig 3).

Many viruses, including enteroviruses, are known to trigger ER stress and activate related signal pathways during their infection, which have profound but complex implications for virus replication and pathogenesis [12,33]. Activation of UPR will restrict virus replication by preventing protein translation, enhancing protein degradation or activating anti-viral immune response; on the other hand, numerous viruses have evolved mechanisms to modulate UPR to survive and propagate in host cells [12,33]. We here demonstrated that enterovirus infection also activates the PERK pathway, but relatively late in the infection cycle. This suggests that while viral infection does induce ER stress, the delayed activation of the PERK pathway, and the resulting global protein translation inhibition, likely has minimal impact on viral infection, since by this point, the majority of viral proteins needed for packaging have already been synthesized. Furthermore, while enteroviruses are sensitive to RIDD pathways during GPI anchor transfer failure, HCV could still replicate efficiently in these cells, suggesting different viruses exploit different mechanisms to modulate ER stress. Our luciferase reporter assays revealed that the enterovirus UTRs are sensitive to the RIDD pathway. In contrast, polyprotein translation mediated by the pTM1 construct was unaffected by PIGS- or PIGK-KO. This pTM1 construct lacks the native viral UTRs, featuring instead a T7 promoter and an EMCV IRES in place of the 5’UTR, and a 3’UTR without a poly (A) tail. Taken together, these findings indicate that the enteroviral 5’UTR is a primary target of RIDD, while the poly (A) tail might function to confer stability to the vRNA [44]. The enterovirus UTR regions were found to be sufficient to mediate this RIDD when flanking a luciferase reporter. Interestingly, most RIDD-targeted mRNAs have stem-loop structures resembling XBP1 mRNA, with endoribonuclease cleavage around a conserved CNGCAGN motif in mammalian cells [45,46]. Enteroviruses employ IRES to initiate vRNA translation in host cells, which are composed of stem-loop structures of varied sizes [47]. All three enteroviruses employed in this study bear a CTGCAG sequence in domain V of the 5’UTR region, but conserved sequence was not found in the 3’UTR region. However, non-canonical, motif-independent cleavage was also described [46]. Therefore, the specific cleavage site of vRNA in PIG-related gene-depleted cells remains under investigation.

## Materials and methods

### Cells

All cell cultures were maintained at 37°C in a humidified 5% CO_2_ incubator. 293T, RD and Huh7 cells were obtained from the American Type Culture Collection (ATCC) and cultured in Dulbecco’s Modified Eagle Medium (DMEM; Gibco, USA) supplemented with 10% (v/v) fetal bovine serum (FBS; OPCEL, China), 100 U/mL penicillin, 1 μg/mL streptomycin (Beyotime Biotechnology, China). CRISPR KO cells were maintained with 2μg/mL puromycin, and RD cells stably expressing T7 polymerase were cultured with 600 μg/mL G418 selection.

### Viruses and viral titration

Parental EV71 and CVB5 viruses as well as the subgenomic replicon system, the NanoLuc reporter viruses EV71-NanoLuc and CVB5-NanoLuc have been described in ref [48,49]. Echovirus 7 was obtained from Xi’an Municipal Center for Disease Control and Prevention. Viruses were propagated and titrated in RD cells. The subgenomic replicon of Echo7 virus was constructed with similar methods as described in [48]. The HCV SGR has been described in ref [50].

All enteroviruses were titrated on RD cells with serially diluted virus infection in 96-well plates. TCID_50_ were calculated by counting the wells with CPE using the Reed–Muench method [51].

### *In vitro* transcription and RNA modification

Viral SGRs or human codon-optimized Renilla luciferase (hRluc) gene were cloned in pTOPO (for hRluc, EV71 or Echo7) or pRBCU (for CVB5) downstream of T7 promoter. *In vitro* transcription and RNA transfection were carried out as previously described [23]. Briefly, Constructs were linearized, purified with phenol–chloroform–isoamyl alcohol and *in vitro* transcribed with HiScribe T7 High Yield RNA Synthesis Kit (NEB, Ipswich, MA). When applicable, RNA was capped with Vaccinia Capping system (NEB) or modified with E.coli Poly (A) Polymerase (NEB). All RNAs are transfected into cells with TransIT-mRNA transfection kit (Mirus, Madison, WI) according to manufacturer’s instructions.

### Plasmids and reagents

The cDNAs encoding PIGS, PIGK, CD55, IRE1α, Sec61β, XBP1, ATF6, IRE1 were obtained by reverse transcription (RT) of total RNA from Huh 7 cells, followed by PCR using the specific primers listed in S2 Table. Sequences encoding the 1–40 aa residuals of SPARC, 5 XATF6 promoter fused with c-fos minimal sequence were synthesized by GENEWIZ, Suzhou, China. Where applicable, silent mutations were introduced into genes to render them resistant to sgRNA targeting. All constructs were subcloned into a lentiviral expression vector [23] with 2X MultiF Seamless Assembly Mix (Abclonal, Woburn, MA). All constructs were confirmed by sequencing.

### Lentiviral vector preparation and transduction

Vesicular stomatitis virus glycoprotein G (VSV-G) pseudotyped lentiviruses were produced as previously reported [38,52]. sgRNA or shRNA sequences used in this study were listed in S3 Table. RD cells were transduced with lentiviral particles prepared above and stable cell lines were obtained by puromycin selection (for sgRNA or shRNA) or blasticidin S selection (for overexpression constructs).

### CRISPR screening

A pooled CRISPR screen was carried out as described in ref [52,53]. Briefly, GeKOv2 plasmid library from Addgene was used to generate VSV-G -pseudotyped lentivirus as described above. RD cells were transduced with lentiviral pools at MOI = 0.3 and selected with puromycin at 2μg/ml for a week. Cells were then infected with Echo7 at MOI = 0.1 for 2 weeks and genome from survival cells were extracted with Quick-DNA Midi kit (Zymo Research, Irvine, CA). The integrated sgRNAs were amplified using PCR, and next generation sequencing was performed at Oebiotech (Shanghai, China) on a MiSeq instrument (Illumina, CA). Data were analyzed with the MAGeCK algorithm [54].

### Virus infection and cell viability test

Cells were infected with Echo7, EV71 or CVB5 at MOI = 1.0 (unless stated otherwise). 18–20 h.p.i cells were harvested for immunoblotting, q-RT-PCR or cell viability evaluation. For cell viability, cells were washed once with PBS and tested with Cell Titer Glo (Promega Corporation, Madison, WI) according to manufacturer’s instruction. Luminescence signals were measured using BioTek Neo2 microplate reader.

### Immunoblotting

Cells treated as indicated were harvested and lysed with 1 × LDS-loading buffer (Thermo Fisher Scientific). SDS-PAGE were carried out followed by transferring to a PVDF membrane. The blots were then probed with indicated primary antibodies at a dilution of 1:1000 (unless otherwise indicated). Following TBST wash, corresponding secondary antibodies were added and blots were visualized using enhanced chemiluminescence reagent (Mishushengwu, Xi’an, China) with FUSION SOLO 6S (Vilber, Paris, France).

### Viral RNA quantification and q-RT-qPCR

For viral RNA analysis and knockdown efficiency determination, total cellular RNA was extracted using the FOREGENE kit (Chengdu, China) according to manufacturer’s instruction. cDNA was reverse transcribed with ABScript III RT Master Mix (Abclonal, Woburn, MA) and qPCR was performed with SYBR Green PCR mix (GenStar, Beijing, China). The comparative CT method (ΔΔCT) was used to measure the transcript levels of each target gene. The specific primers used to analyze enterovirus and PGAP3 transcripts are described in the S4 Table.

For viral RNA stability test, Control, PIGS- or PIGK-KO RD cells were transfected with EV71-SGR (GND) with TransIT-mRNA transfection kit (Mirus, Madison, WI) according to manufacturer’s instructions. 2 hrs later, cells were replenished with fresh medium. Cellular total RNA was harvested at 4, 6, 8, 10 hrs post-transfection, viral RNAs were determined with q-RT-PCR as described above. vRNAs were normalized to 4 hr data and plotted against time.

### Flow cytometry analysis

To determine the surface expression of GPI anchored CD55 or CD55/TM, cells were incubated with a FITC-conjugated anti-CD55 antibody (SinoBiological, Beijing, China) for 1hr on ice. Cells were then washed three times with PBS and analyzed using a CytoFLEX cytometer (Beckman Coulter, IN). Data analysis was performed using FlowJo.

### Luciferase assay

Cells were either infected with EV71-NanoLuc or CVB5-NanoLuc or transfected with SGRs containing *Renilla* luciferase reporter for indicated time. Nano luciferase and Renilla luciferase were measured with Nano-Glo® or Renilla luciferase assay system from Promega (Madison, WI) following the manufacturer’s instructions. Luminescence signals were measured using BioTek Neo2 microplate reader.

### Virus attachment and internalization assay

Viral binding and internalization assays were performed as previously described [52]. Briefly, control, PIGS- or PIGK KO cells, as well as CD55/TM complemented cells were incubated with Echo7 (MOI = 10) at 4°C for 1 h (attachment assay) or incubated at 4°C for 1 h followed by 1 h of incubation at 37°C (internalization assay). Cells were then washed extensively with ice-cold PBS, and viral RNA was determined by q-RT-PCR as described above.

### Confocal microscopy

Cells were seeded on poly-D-lysine coated coverslips and treated or infected with virus as indicated. The cells were fixed with ice-cold methanol for 10 min and then blocked with 10% FBS at room temperature for 1 h. Anti-PIGK (1:100), anti-HA (1:200), anti-dsRNA (1:300), anti-EV71 3AB (1:100), anti-CALR (1:100), anti-GM130 (1:100), anti-TGN46 (1:100), anti-HSP60 (1:100) were used for immunostaining. After 1 hr incubation at room temperature, Alexa Fluor 488 or Alexa Fluor 555 conjugated secondary antibodies (1:500) were applied for another 1 hr at room temperature. Cells were mounted in ProLong Gold Antifade mountant after DAPI staining. All laser-scanning images were acquired on an Olympus FV3000 confocal microscope.

### Membrane flotation assay

Membrane flotation was carried out as previously described [23,24] with modifications. Briefly, RD cells, or EV71 infected RD cells were homogenized with a ball-bearing homogenizer (Isobiotec, Heidelberg, Germany) and supernatants (H) were centrifuged at 3000 × g 1 hr at 4°C to get water soluble fraction (WS), while the pellets were treated with 0.05% ice-cold NP-40. After another centrifugation at 3000 × g for 30min to get detergent soluble fraction (DS), the insoluble pellets were overlaid to Optiprep gradient (STEMCELL Technologies, Canada) and centrifuged with CS150 NX micro Ultracentrifuge (Eppendorf Himac Technologies) using a S52ST swinging bucket rotor at 45,000 rpm for 4 h at 4 °C. After centrifugation, fractions were collected from top and proteins were concentrated by TCA precipitation and analyzed with immunoblotting as described above. The band density was quantified with Image J and fractional protein percentage was determined from the band density and volume of the fraction relative to the total input protein.

### Transmission electron microscopy

Control, PIGS- or PIGK KO cells stably expressing T7 RNA polymerase were seeded in 10 cm cell culture dish and transfected with pTM1 (2A-3D). Cells were harvested 48 hrs later and analyzed with immunoblotting to determine the expression of viral NS. For TEM, cell pellets were first fixed with Gluta fixing solution (Solarbio Life Science, Beijing, China) at 4 °C, followed by post fixed in 1% osmium tetroxide and dehydrated in a graded ethanol series. Samples were then embedded in Epon and ultra-thin sections were prepared and collected onto copper grids and post stained with uranyl acetate and lead citrate. The sections were viewed on a Hitachi H-7650 transmission electron microscope.

### RNA immunoprecipitation

RIP experiment was carried out as previously described [55]. Briefly, RD cells stably transduced with endoribonuclease dead mutant of IRE1 were transfected with 5ULuc3U or CLucPA RNA as described above. 4 hrs later, cells were lysed with IP buffer (25 mM Tris pH 7.4, 150 mM NaCl, 1% NP-40, 1 mM EDTA) supplemented with protease inhibitor and RNase inhibitor. After centrifuge at 1200 × g for 5min at 4°C, supernatants were collected and “Input” was saved. Supernatant was then added with 4 μg anti-phospho-IRE1 antibody and kept on ice for 1 hr with occasional agitation. 30 μl protein-A/G magnetic beads (Selleckchem, Houston, TX) were then used to precipitate RNA. Samples were then assayed with immunoblotting or q-RT-PCR as described above. A pTopo-RLuc plasmid was serially diluted to generate a standard curve for quantification of RNA.

### Quantification and statistical analysis

Data are presented as mean ± sd. Statistical analyses were carried out with GraphPad Prism 9. Statistical comparisons between two groups were performed using unpaired Student’s t-test. For comparisons across more than two groups, a one-way ANOVA was employed, followed by Dunnett’s post-hoc test. Ns, not significant; *, p < 0.05; **, p < 0.01; ***, p < 0.001.

## Supporting information

S1 Fig GPI-related genes are required for enterovirus infection. (A) Diagram of pooled CRISPR screening against Echo7 infection. (B) Control, PIGS- or PIGK-KO cells were infected with Echo7 virus at MOI = 1 and viral RNA was quantified with q-RT-PCR. ***, p < 0.001, compared to control cells. (C) Control, PIGS- or PIGK-KO cells were either uninfected or infected with Echo7 virus at MOI = 1 and cell viability was determined 20 h.p.i. ***, p < 0.001, compared to uninfected cells. (D) PIGS KO (left panels) or PIGK KO (right panels) cells were transduced with lentiviral vector expressing sgRNA-resistant PIGS or PIGK and infected with Echo 7 at MOI = 1 and VP3 expression was determined at 20 h.p.i. (E-G) PIGT (E), PIGH (F) or GPAA1 (G) knockout cells were infected with Echo 7 at MOI = 1 and cell viability was determined at 20 h.p.i. *, p < 0.05; **, p < 0.01; ***, p < 0.001, compared to uninfected cells. (H-I) Control, PIGS- or PIGK-KO cells were infected with EV71 (H) or CVB5 (I) at MOI = 1 and viral RNA was quantified with q-RT-PCR. ***, p < 0.001, compared to control cells. (J-K) Control, PIGS- or PIGK-KO cells were either uninfected or infected with EV71 (J) or CVB5 (K) at MOI = 1 and cell viability was determined 20 h.p.i. *, p < 0.05; **, p < 0.01; ***, p < 0.001, compared to uninfected cells.
(TIF)

S2 Fig CD55 is not required for EV71 and CVB5 infection. (A) Control, CD55-KO or CD55-KO cells supplemented with sgRNA-resistant CD55 were infected with EV71, Echo7, or CVB5 and cell viability was determined. ***, p < 0.001, compared to uninfected cells. (B) Control, CD55-KO or CD55-KO cells supplemented with CD55/GPI or CD55/TM were either untreated or treated with 1 U/ml PLC for 30min before they were infected with Echo7, and cell viability was determined at 20 h.p.i. *, p < 0.05; ***, p < 0.001, compared to uninfected cells.
(TIF)

S3 Fig GPI genes are required for enterovirus replication. (A) RD cells were mock treated or treated with 1 U/ml PLC for 30 min before they were infected with EV71, Echo7, or CVB5 at MOI = 1 and cell viability was determined. ***, p < 0.001, compared to uninfected cells. (B-C) Upper panels: Diagrams of CVB5-SGR (GND) (B) or Echo7-SGR (GND) (C). Lower panels: control, PIGS-KO or PIGK-KO cells were transfected with CVB5-SGR (GND) (B) or Echo7-SGR (GND) (C) and luciferase was monitored. **, p < 0.01; ***, p < 0.001, compared to control cells. (D) Control, PIGS-, PIGK KO cells were transfected with plasmid encoding luciferase and luciferase activity was measured 24 hrs post-transfection. ns, not significant, compared to control.
(TIF)

S4 Fig GPI genes are required for enterovirus RO biogenesis. (A) Representative electron microscopy image of RD/T7 cells transfected with pTM1 (2A-3D). (B-E) PIGS-KO (B, C) or PIGK-KO cells (D, E) stably expressing T7 polymerase were transfected with pTM1 (2A-3D) and electron microscopy images were shown. Asterisks indicate multi-membrane vesicles. (F) Quantification of the number of membrane vesicles in pTM1 (2A-3D) transfected control, PIGS- or PIGK KO cells stably expressing T7 polymerase. Each data point corresponds to the vesicles count from a single image with an area of 10μm2. ***, p < 0.001, compared to control cells.
(TIF)

S5 Fig GPI genes were not required for Golgi integrity. (A) Control, PIGS- or PIGK-KO cells were immunoblotted with anti-GM130 (upper panels) or anti-TGN46 (lower panels) with nuclei counterstaining. Bar, 10 μm. (B) Control, PIGS- or PIGK-KO cells were either untreated (upper panel) or treated with 150 μM oleic acid (OA) for 16 hrs before LDs were visualized with Bodipy 493/503 and images were taken with confocal microscope. Bar, 10 mm. (C) Fluorescence intensity of cells treated as in (B) was quantified with microplate reader. ns, not significant, compared to control. (D) The overall amount of lipids in control, PIGS- or PIGK-KO cells were quantified with Lipid Quantification Kit from Cell Biolabs and normalized to control cells. ns, not significant, compared to control.
(TIF)

S6 Fig PERK or ATF6 pathway was not activated in PIGS- or PIGK-KO cells. (A) Control, PIGS-, PIGK KO cells were immunoblotted with indicated antibodies. Control cells treated with 0.15 μM TG for 6 hrs were used as positive control. (B-E) Transcriptional analysis of CHOP, GADD34, BIP or PID4A expression in PIGS-, PIGK-KO cells. Cells treated with 0.15 μM TG for 6 hrs were used as positive control. ns, not significant; ***, p < 0.001, compared to control. (F) Control, PIGS-, PIGK KO cells were transfected with plasmid encoding luciferase downstream of 5 × ATF6 promoter fused with c-fos minimal sequences. Luciferase was determined 24 hrs post-transfection. ns, not significant; *, p < 0.05; ***, p < 0.001, compared to control. (G) Control, PIGS-KO or PIGK-KO cells were either mock treated or treated with 20 μM 4μ8C for 16 hrs before they were infected with NanoLuc-EV71. Luciferase activities were monitored at indicated time points. Inset, enlargement of the first 4 hrs. ns, not significant; **, p < 0.01; ***, p < 0.001, 4μ8C treated values compared to nontreated values.
(TIF)

S7 Fig Knockout of PIGB or PIGO does not activate RIDD. (A) Validation of PIGC (upper panels) and PIGH (lower panels) knockout in RD cell lines. The sgRNA target sites for each gene are highlighted in red, aligned with the mutant alleles that show frameshift mutations. Sequencing chromatograms confirmed the insertion of an extra ‘T’ nucleotide in the PIGC and PIGH mutants. (B) Control, PIGB KO (upper panels), or PIGO KO (lower panels) cells were infected with Echo7 virus at MOI = 1 for 20 hrs and viral VP3 expression was detected with immunoblotting. sgRNAs underlined were chosen for downstream experiments. (C) Validation of PIGB (upper panels) and PIGO (lower panels) knockout in RD cell lines. The sgRNA target sites for each gene are highlighted in red, aligned with the mutant alleles that show frameshift mutations. Sequencing chromatograms confirmed the deletion of four or eight nucleotides in the PIGB or PIGO mutant, respectively. Note the reverse sequencing of PIGB was shown. (D) Control, PIGB KO, or PIGO KO cells transfected with IRE1-GFP and evaluated with confocal microscopy. TG was used as a positive control. Bar, 10μm. (E-F) Control, PIGB KO, or PIGO KO cells were immunoblotted with indicated antibodies (E) or tested for spliced XBP1 expression with q-RT-PCR (F). ns, not significant; ***, p < 0.001, compared to values in control group. (G) Indicated cells were transfected with XBP1-DDBD-NL and luciferase was measured. Cell viability was measured with Cell Titer Glo. **, p < 0.01; ***, p < 0.001, compared to values in control group. (H-I) SCARA (H) or Col6A1 (I) expressions in indicated cells were determined with q-RT-PCR. ns, not significant; *, p < 0.05; **, p < 0.01, compared to values in control group. (J) Indicated cells were transfected with GFP or SPARC-GFP and the expression was determined with immunoblotting.
(TIF)

S8 Fig Enterovirus infection activates PERK pathway. (A) RD cells infected with EV71, Echo7 or CVB5 (MOI = 1) for 20 hrs and supernatants were collected for virus titer determination. ns, not significant among different groups. (B-D) RD cells infected with EV71, Echo7 or CVB5 (MOI = 1) were tested for BLOC1S1 (B), PDIA4 (C) or SEL1L (D) expression with q-RT-PCR. ns, not significant, compared to values in control group. (E). RD cells transfected with 5 × ATF6 luciferase reporter plasmid were infected with EV71, Echo7 or CVB5 and luciferase activity was measured. *, p < 0.05; ***, p < 0.001, compared to values in control group.
(TIF)

S9 Fig Luciferase or GFP expression was not altered in PIGS- or PIGK-KO cells. (A) Representative images of control, PIGS-, PIGK-KO cells were transfected with plasmid encoding GFP. Bar, 20mm. (B) Control, PIGS-, PIGK KO cells were transfected with plasmid encoding GFP, and GFP expression was determined with immunoblotting. (C) RD cells stably expressing IRE (K907A) were immunoprecipitated with anti-phospho-IRE1 and subjected to immunoblotting. (D-E) Serially diluted pTopo-RLuc plasmids were quantitated with qPCR and the amplification curves (D) and standard curve generated (E) were shown. (F) RD cells stably expressing IRE (K907A) were transfected with CLucPA or 5ULuc3U RNA for 4 hrs before they were immunoprecipitated with indicated antibodies and the precipitated RNAs were quantitated with q-RT-PCR and shown as percentage of input RNA. ns, not significant; ***, p < 0.001, compared to values in CLucPA group.
(TIF)

S1 Table CRISPR Screening Gene Enrichment list. (XLSX)

S2 Table Primers used for constrution of plasmids. (XLSX)

S3 Table sgRNA or shRNA used in this study (XLSX)

S4 Table qPCR primers used in this study. (XLSX)
