# Supplementary figures and images for: Disrupted glycosylphosphatidylinositol anchoring induces ER stress and restricts enterovirus infection
Source: PLoS Pathog. 2025 Nov 18;21(11):e1013685. doi: 10.1371/journal.ppat.1013685 (PMC12626292; doi:10.1371/journal.ppat.1013685)

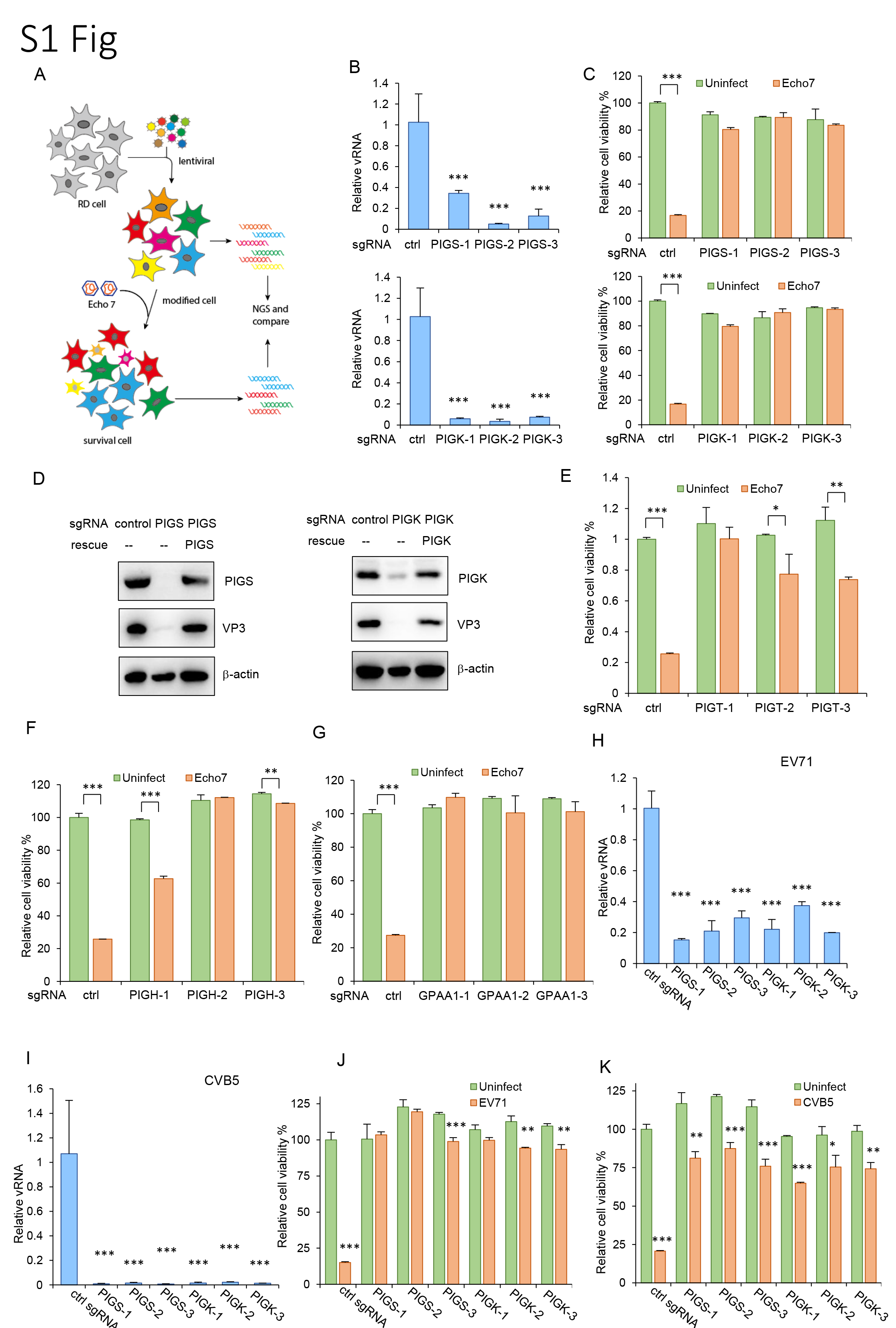

Supplement: S1 Fig — (A) Diagram of pooled CRISPR screening against Echo7 infection. (B) Control, PIGS- or PIGK-KO cells were infected with Echo7 virus at MOI = 1 and viral RNA was quantified with q-RT-PCR. ***, p < 0.001, compared to control cells. (C) Control, PIGS- or PIGK-KO cells were either uninfected or infected with Echo7 virus at MOI = 1 and cell viability was determined 20 h.p.i. ***, p < 0.001, compared to uninfected cells. (D) PIGS KO (left panels) or PIGK KO (right panels) cells were transduced with lentiviral vector expressing sgRNA-resistant PIGS or PIGK and infected with Echo 7 at MOI = 1 and VP3 expression was determined at 20 h.p.i. (E-G) PIGT (E), PIGH (F) or GPAA1 (G) knockout cells were infected with Echo 7 at MOI = 1 and cell viability was determined at 20 h.p.i. *, p < 0.05; **, p < 0.01; ***, p < 0.001, compared to uninfected cells. (H-I) Control, PIGS- or PIGK-KO cells were infected with EV71 (H) or CVB5 (I) at MOI = 1 and viral RNA was quantified with q-RT-PCR. ***, p < 0.001, compared to control cells. (J-K) Control, PIGS- or PIGK-KO cells were either uninfected or infected with EV71 (J) or CVB5 (K) at MOI = 1 and cell viability was determined 20 h.p.i. *, p < 0.05; **, p < 0.01; ***, p < 0.001, compared to uninfected cells. (TIF) [file ppat.1013685.s001.tif]

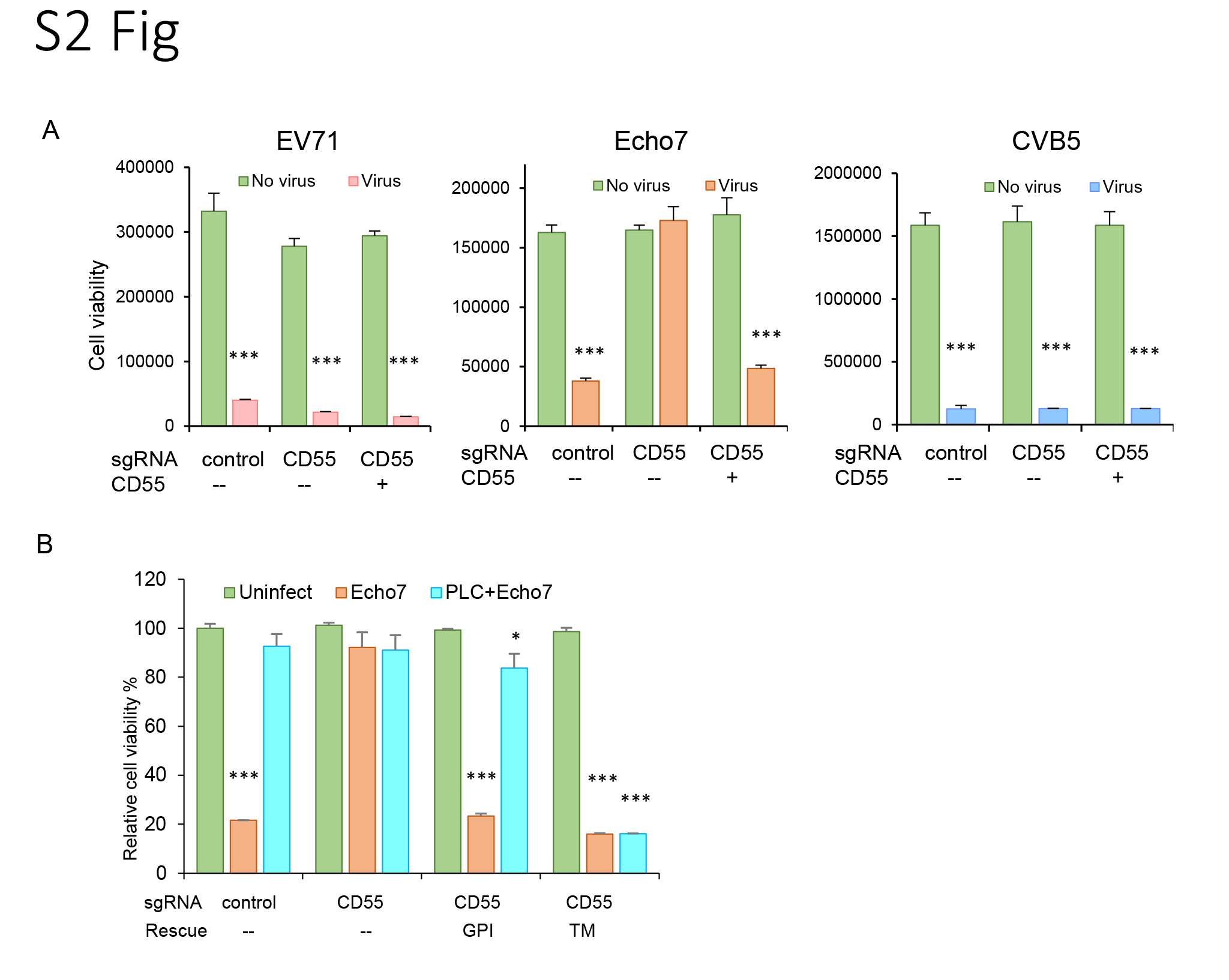

Supplement: S2 Fig — (A) Control, CD55-KO or CD55-KO cells supplemented with sgRNA-resistant CD55 were infected with EV71, Echo7, or CVB5 and cell viability was determined. ***, p < 0.001, compared to uninfected cells. (B) Control, CD55-KO or CD55-KO cells supplemented with CD55/GPI or CD55/TM were either untreated or treated with 1 U/ml PLC for 30min before they were infected with Echo7, and cell viability was determined at 20 h.p.i. *, p < 0.05; ***, p < 0.001, compared to uninfected cells. (TIF) [file ppat.1013685.s002.tif]

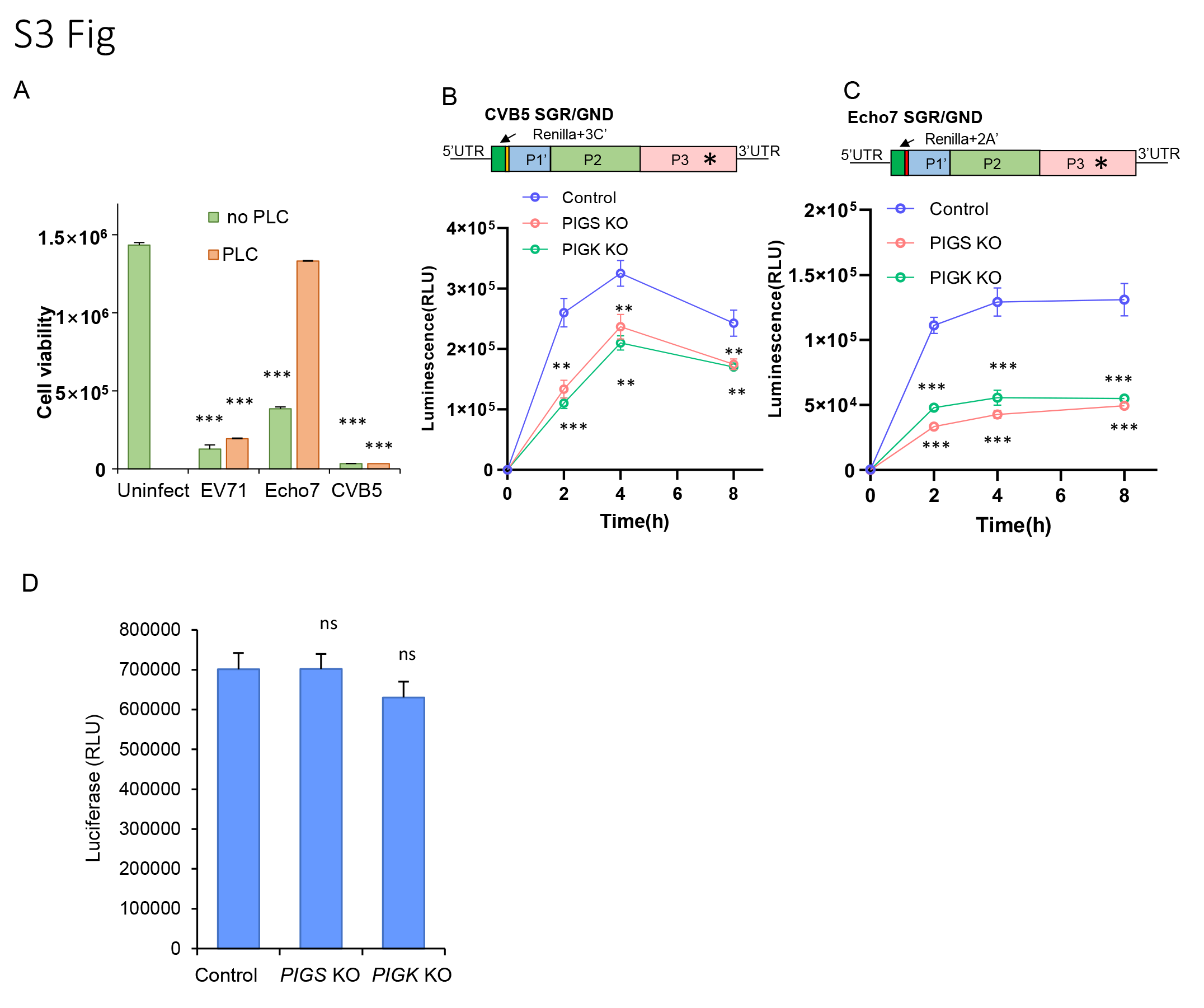

Supplement: S3 Fig — (A) RD cells were mock treated or treated with 1 U/ml PLC for 30 min before they were infected with EV71, Echo7, or CVB5 at MOI = 1 and cell viability was determined. ***, p < 0.001, compared to uninfected cells. (B-C) Upper panels: Diagrams of CVB5-SGR (GND) (B) or Echo7-SGR (GND) (C). Lower panels: control, PIGS-KO or PIGK-KO cells were transfected with CVB5-SGR (GND) (B) or Echo7-SGR (GND) (C) and luciferase was monitored. **, p < 0.01; ***, p < 0.001, compared to control cells. (D) Control, PIGS-, PIGK KO cells were transfected with plasmid encoding luciferase and luciferase activity was measured 24 hrs post-transfection. ns, not significant, compared to control. (TIF) [file ppat.1013685.s003.tif]

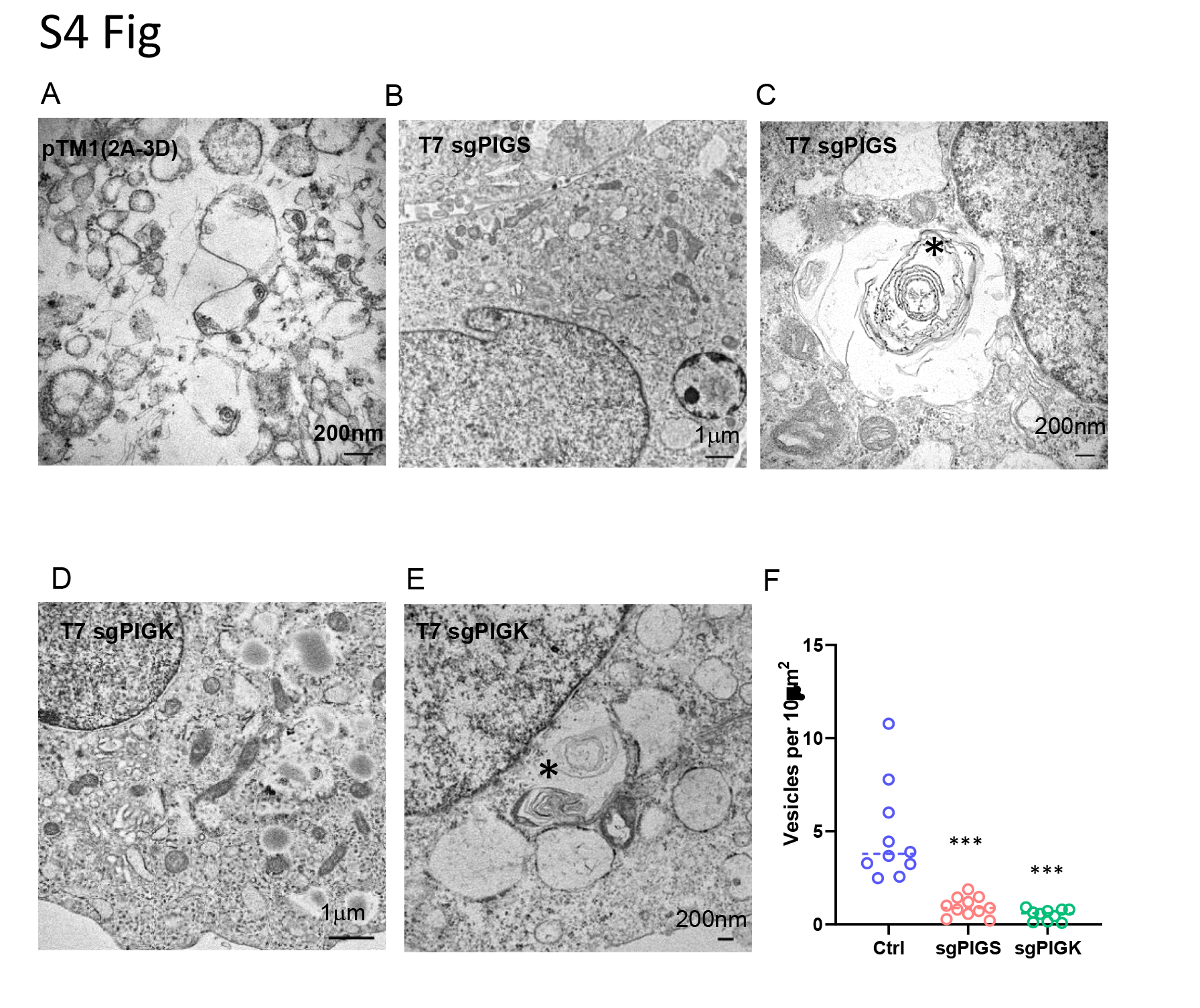

Supplement: S4 Fig — (A) Representative electron microscopy image of RD/T7 cells transfected with pTM1 (2A-3D). (B-E) PIGS-KO (B, C) or PIGK-KO cells (D, E) stably expressing T7 polymerase were transfected with pTM1 (2A-3D) and electron microscopy images were shown. Asterisks indicate multi-membrane vesicles. (F) Quantification of the number of membrane vesicles in pTM1 (2A-3D) transfected control, PIGS- or PIGK KO cells stably expressing T7 polymerase. Each data point corresponds to the vesicles count from a single image with an area of 10μm2. ***, p < 0.001, compared to control cells. (TIF) [file ppat.1013685.s004.tif]

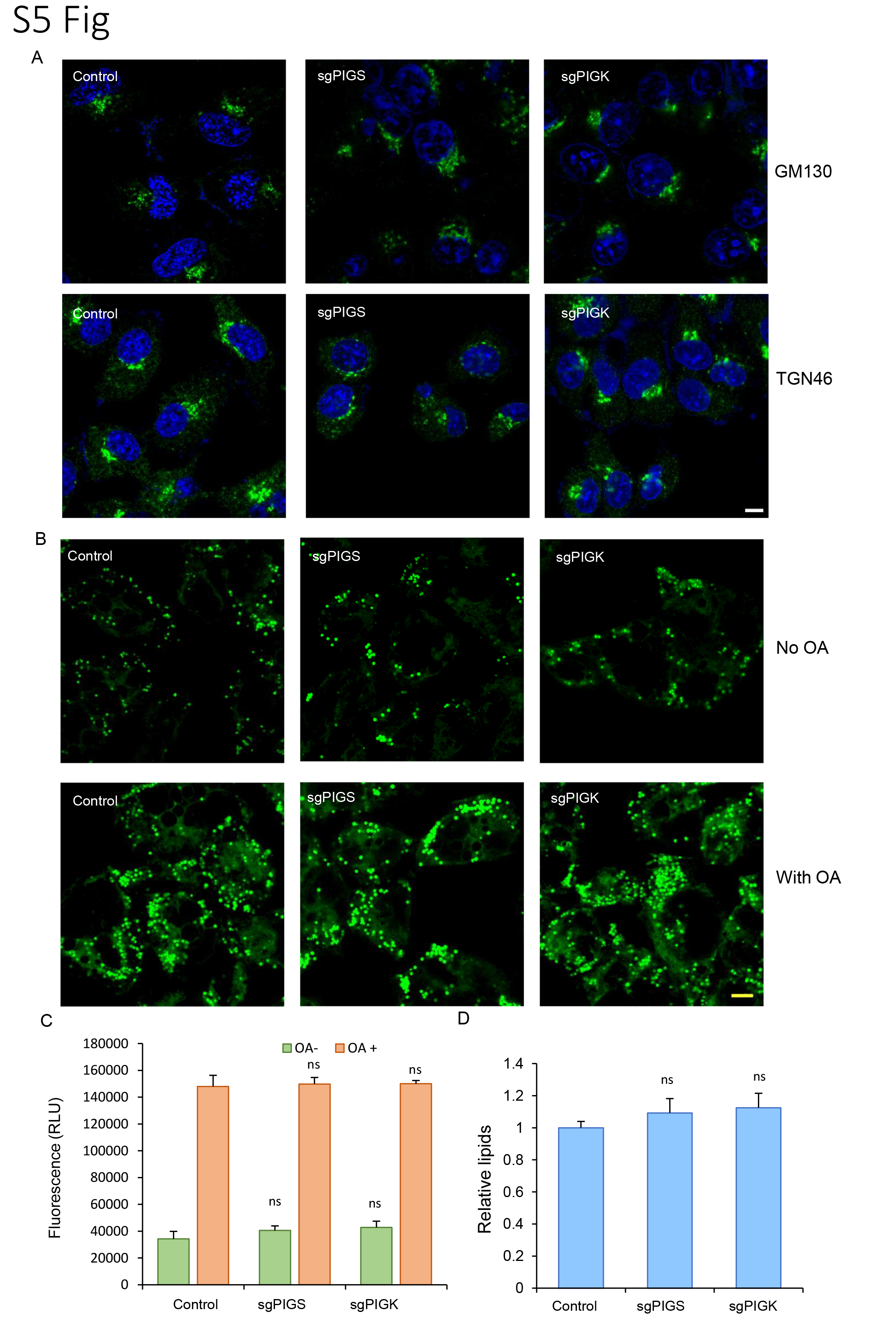

Supplement: S5 Fig — (A) Control, PIGS- or PIGK-KO cells were immunoblotted with anti-GM130 (upper panels) or anti-TGN46 (lower panels) with nuclei counterstaining. Bar, 10 μm. (B) Control, PIGS- or PIGK-KO cells were either untreated (upper panel) or treated with 150 μM oleic acid (OA) for 16 hrs before LDs were visualized with Bodipy 493/503 and images were taken with confocal microscope. Bar, 10 mm. (C) Fluorescence intensity of cells treated as in (B) was quantified with microplate reader. ns, not significant, compared to control. (D) The overall amount of lipids in control, PIGS- or PIGK-KO cells were quantified with Lipid Quantification Kit from Cell Biolabs and normalized to control cells. ns, not significant, compared to control. (TIF) [file ppat.1013685.s005.tif]

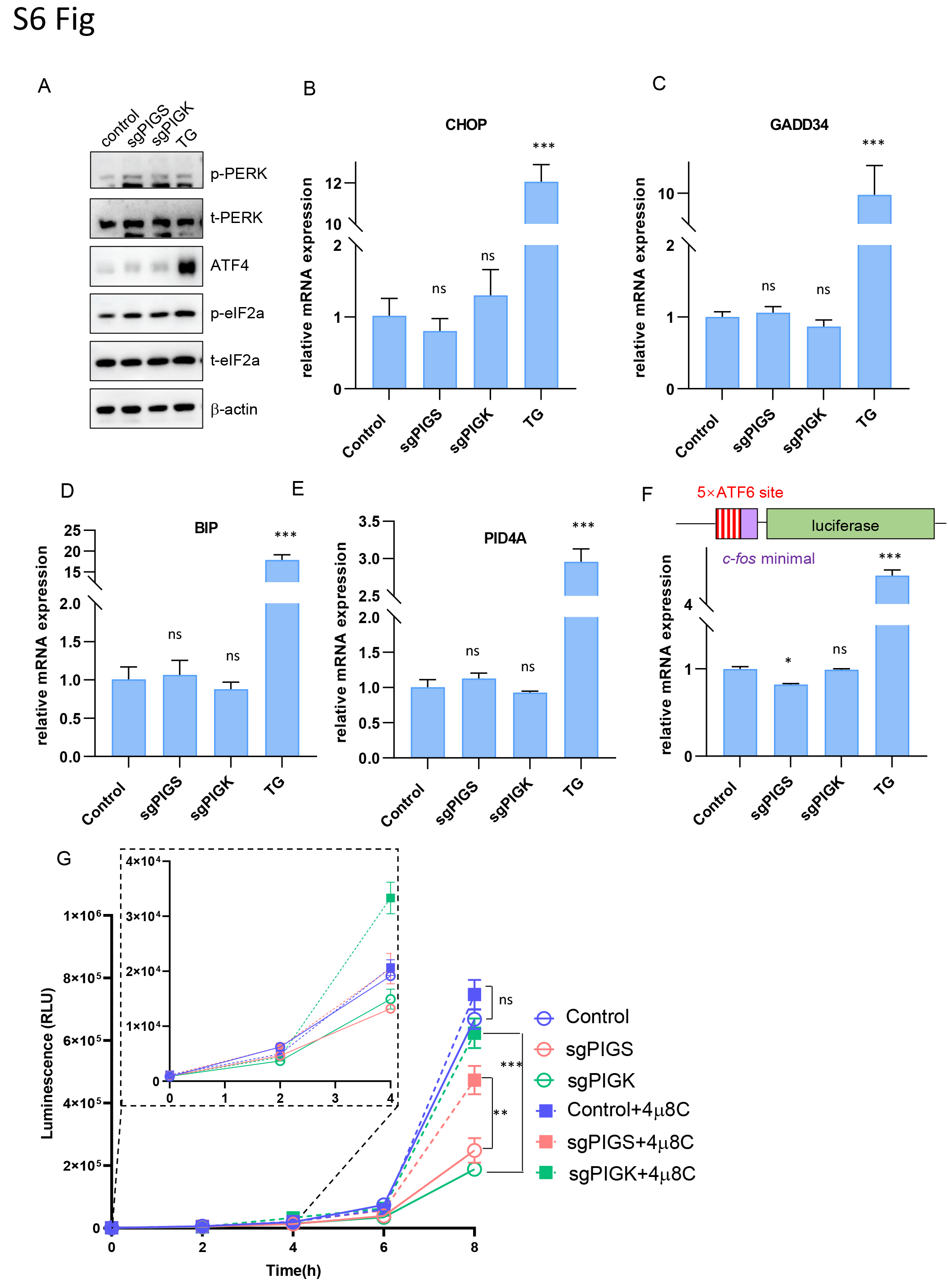

Supplement: S6 Fig — (A) Control, PIGS-, PIGK KO cells were immunoblotted with indicated antibodies. Control cells treated with 0.15 μM TG for 6 hrs were used as positive control. (B-E) Transcriptional analysis of CHOP, GADD34, BIP or PID4A expression in PIGS-, PIGK-KO cells. Cells treated with 0.15 μM TG for 6 hrs were used as positive control. ns, not significant; ***, p < 0.001, compared to control. (F) Control, PIGS-, PIGK KO cells were transfected with plasmid encoding luciferase downstream of 5 × ATF6 promoter fused with c-fos minimal sequences. Luciferase was determined 24 hrs post-transfection. ns, not significant; *, p < 0.05; ***, p < 0.001, compared to control. (G) Control, PIGS-KO or PIGK-KO cells were either mock treated or treated with 20 μM 4μ8C for 16 hrs before they were infected with NanoLuc-EV71. Luciferase activities were monitored at indicated time points. Inset, enlargement of the first 4 hrs. ns, not significant; **, p < 0.01; ***, p < 0.001, 4μ8C treated values compared to nontreated values. (TIF) [file ppat.1013685.s006.tif]

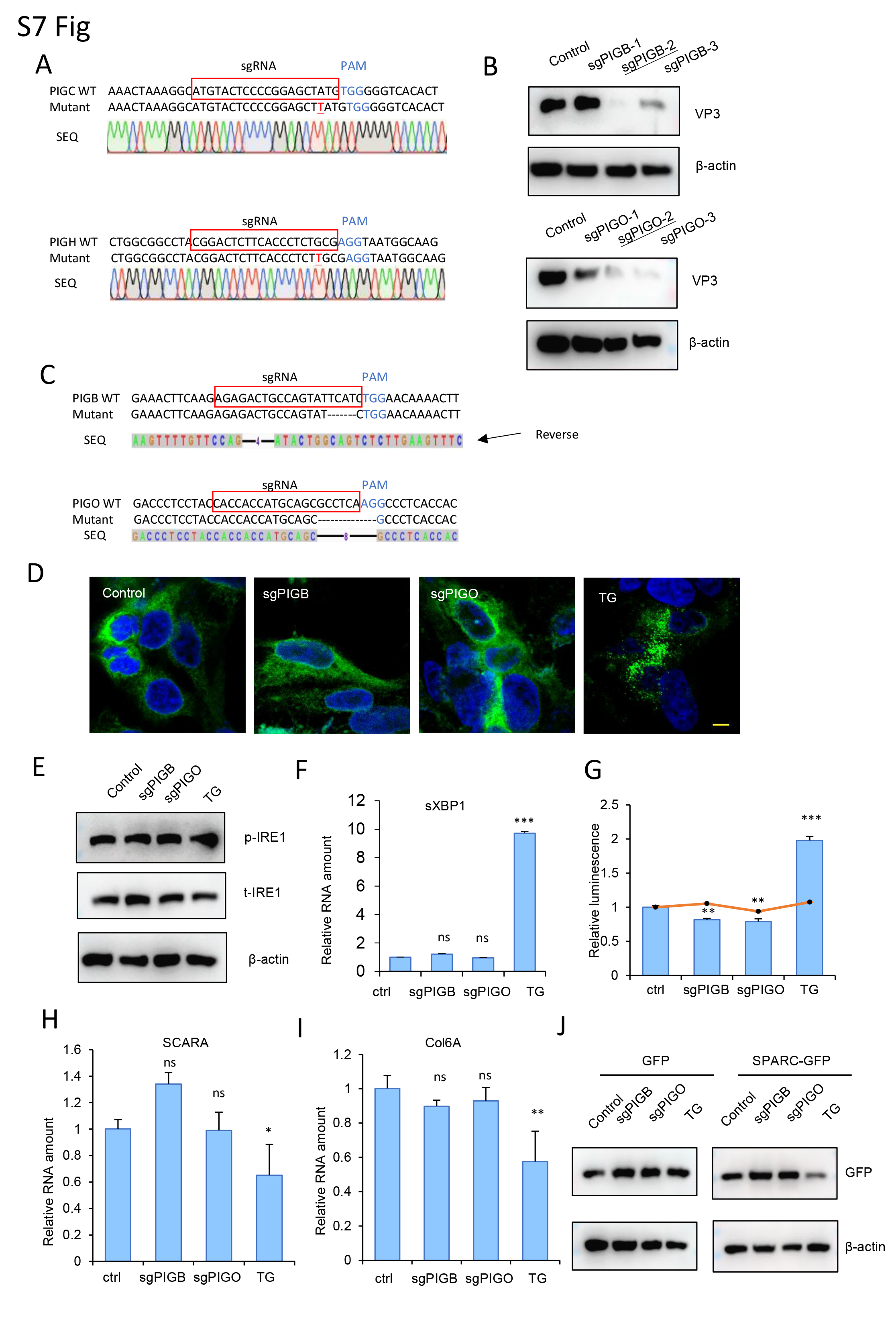

Supplement: S7 Fig — (A) Validation of PIGC (upper panels) and PIGH (lower panels) knockout in RD cell lines. The sgRNA target sites for each gene are highlighted in red, aligned with the mutant alleles that show frameshift mutations. Sequencing chromatograms confirmed the insertion of an extra ‘T’ nucleotide in the PIGC and PIGH mutants. (B) Control, PIGB KO (upper panels), or PIGO KO (lower panels) cells were infected with Echo7 virus at MOI = 1 for 20 hrs and viral VP3 expression was detected with immunoblotting. sgRNAs underlined were chosen for downstream experiments. (C) Validation of PIGB (upper panels) and PIGO (lower panels) knockout in RD cell lines. The sgRNA target sites for each gene are highlighted in red, aligned with the mutant alleles that show frameshift mutations. Sequencing chromatograms confirmed the deletion of four or eight nucleotides in the PIGB or PIGO mutant, respectively. Note the reverse sequencing of PIGB was shown. (D) Control, PIGB KO, or PIGO KO cells transfected with IRE1-GFP and evaluated with confocal microscopy. TG was used as a positive control. Bar, 10μm. (E-F) Control, PIGB KO, or PIGO KO cells were immunoblotted with indicated antibodies (E) or tested for spliced XBP1 expression with q-RT-PCR (F). ns, not significant; ***, p < 0.001, compared to values in control group. (G) Indicated cells were transfected with XBP1-DDBD-NL and luciferase was measured. Cell viability was measured with Cell Titer Glo. **, p < 0.01; ***, p < 0.001, compared to values in control group. (H-I) SCARA (H) or Col6A1 (I) expressions in indicated cells were determined with q-RT-PCR. ns, not significant; *, p < 0.05; **, p < 0.01, compared to values in control group. (J) Indicated cells were transfected with GFP or SPARC-GFP and the expression was determined with immunoblotting. (TIF) [file ppat.1013685.s007.tif]

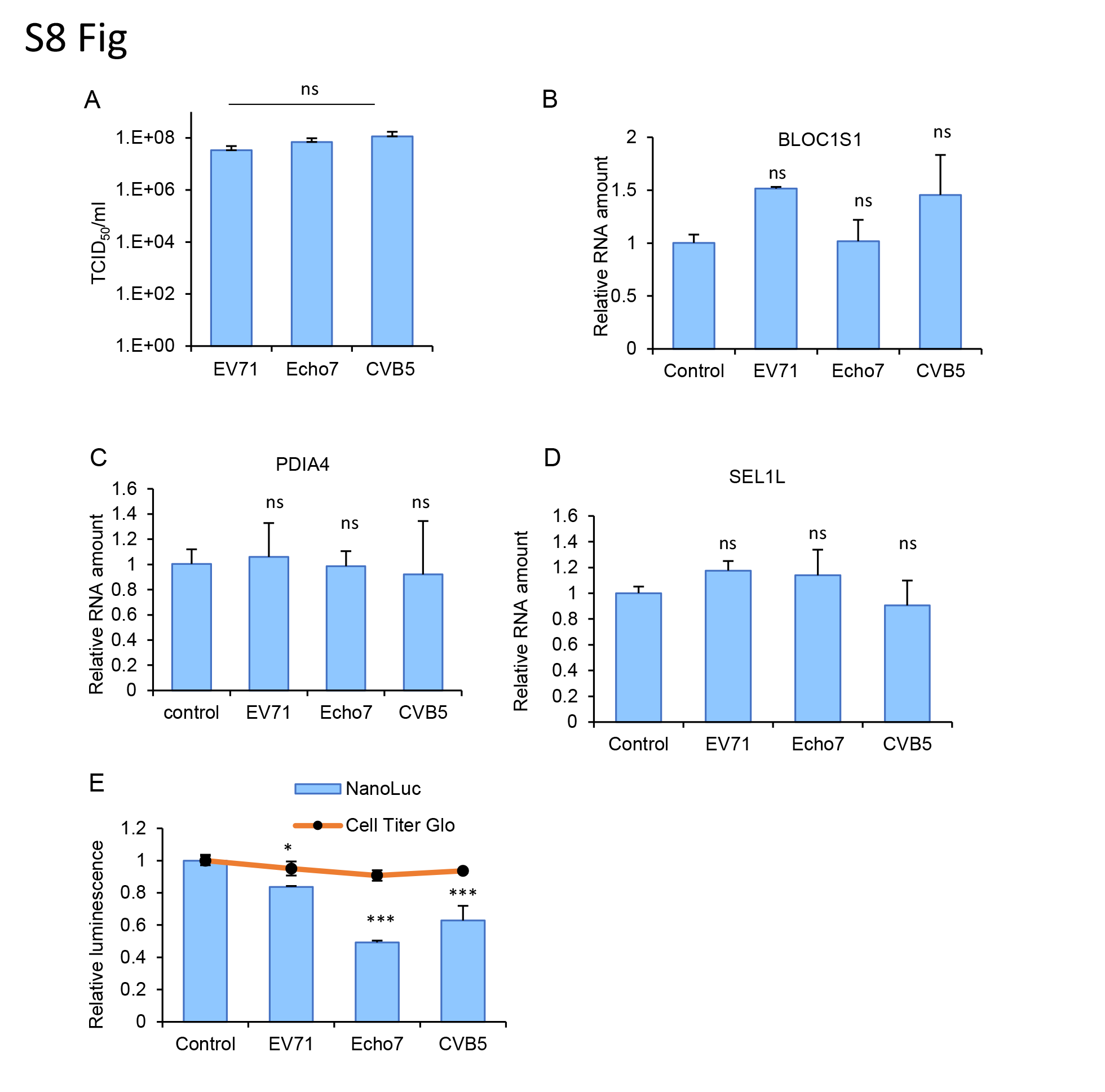

Supplement: S8 Fig — (A) RD cells infected with EV71, Echo7 or CVB5 (MOI = 1) for 20 hrs and supernatants were collected for virus titer determination. ns, not significant among different groups. (B-D) RD cells infected with EV71, Echo7 or CVB5 (MOI = 1) were tested for BLOC1S1 (B), PDIA4 (C) or SEL1L (D) expression with q-RT-PCR. ns, not significant, compared to values in control group. (E). RD cells transfected with 5 × ATF6 luciferase reporter plasmid were infected with EV71, Echo7 or CVB5 and luciferase activity was measured. *, p < 0.05; ***, p < 0.001, compared to values in control group. (TIF) [file ppat.1013685.s008.tif]

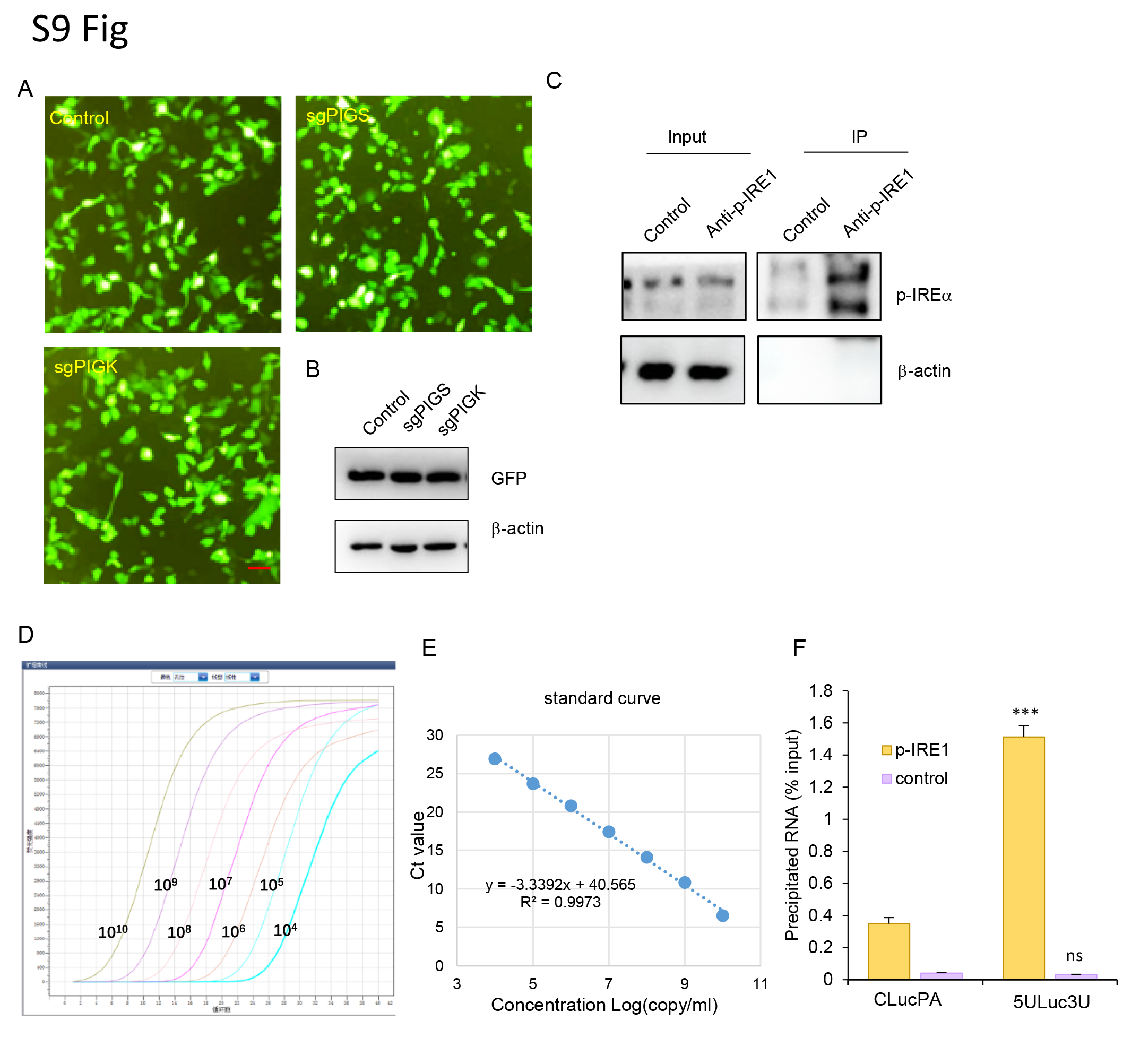

Supplement: S9 Fig — (A) Representative images of control, PIGS-, PIGK-KO cells were transfected with plasmid encoding GFP. Bar, 20mm. (B) Control, PIGS-, PIGK KO cells were transfected with plasmid encoding GFP, and GFP expression was determined with immunoblotting. (C) RD cells stably expressing IRE (K907A) were immunoprecipitated with anti-phospho-IRE1 and subjected to immunoblotting. (D-E) Serially diluted pTopo-RLuc plasmids were quantitated with qPCR and the amplification curves (D) and standard curve generated (E) were shown. (F) RD cells stably expressing IRE (K907A) were transfected with CLucPA or 5ULuc3U RNA for 4 hrs before they were immunoprecipitated with indicated antibodies and the precipitated RNAs were quantitated with q-RT-PCR and shown as percentage of input RNA. ns, not significant; ***, p < 0.001, compared to values in CLucPA group. (TIF) [file ppat.1013685.s009.tif]
